# Genotoxicity and oxidative stress induction by polystyrene nanoparticles in the colorectal cancer cell line HCT116

**DOI:** 10.1371/journal.pone.0255120

**Published:** 2021-07-23

**Authors:** Giulia Vecchiotti, Sabrina Colafarina, Massimo Aloisi, Osvaldo Zarivi, Piero Di Carlo, Anna Poma

**Affiliations:** 1 Department of Life, Health and Environmental Sciences, University of L’Aquila, L’Aquila, Italy; 2 Department of Psychological, Health & Territorial Sciences, University “G. d’Annunzio” of Chieti-Pescara, Chieti, Italy; 3 Center for Advanced Studies and Technology—CAST, University “G. d’Annunzio” of Chieti-Pescara, Chieti, Italy; VIT University, INDIA

## Abstract

The potential risks of environmental nanoparticles (NPs), in particular Polystyrene Nanoparticles (PNPs), is an emerging problem; specifically, the interaction of PNPs with intestinal cells has not been characterized so far. The mechanism by which polystyrene particles are transferred to humans has not yet been clarified, whether directly through ingestion from contaminated food. We evaluated the interaction between PNPs and colorectal adenocarcinoma cells (HCT116). Cells were exposed to different concentrations of PNPs, metabolic activity and the consequent cytotoxic potential were assessed through viability test; we evaluated the PNP genotoxic potential through the Cytokinesis-Block Micronucleus cytome (CBMN cyt) assay. Finally, we detected Reactive Oxygen Species (ROS) production after NPs exposure and performed Western Blot analysis to analyze the enzymes (SOD1, SOD2, Catalase, Glutathione Peroxidase) involved in the cell detoxification process that comes into play during the cell-PNPs interaction. This work analyzes the cyto and genotoxicity of PNPs in the colorectal HCT116 cell line, in particular the potential damage from oxidative stress produced by PNPs inside the cells related to the consequent nuclear damage. Our results show moderate toxicity of PNPs both in terms of ROS production and DNA damage. Further studies will be needed on different cell lines to have a more complete picture of the impact of environmental pollution on human health in terms of PNPs cytotoxicity and genotoxicity.

## Introduction

Plastic is a widespread pollutant that extends from the marine to the human ecosystem throughout the entire globe. Every year over 8 tons of plastic polymers end up at sea [1]. Plastic particles that have an upper size limit of 5 mm or 1 mm are considered microplastics (MPs) whereas nanoplastics (NPPs) may have a higher size limit of 100 nm or 1000 nm [2]. In the environment, plastic undergoes an important and continuous degradation, thus leading to the formation of NPs obtained from the fragmentation of microplastics [3, 4]. NPPs are considered emerging contaminants whose toxic potential is still unknown, while they are widely used in manufacturing and personal care product [5–7].

The European Commission has already presented in 2018 a regulation aimed at limiting plastic waste in the sea [8], the accumulation of which leads to the so-called fragments of garbage in the sea and in the oceans [9]. Fish, clams and various aquatic species have always been one of the major protein sources worldwide, consequently plastic particles can enter the food chain and contaminate foods intended for human use [10]. The accumulation of MP in fish occurs mainly at the level of gills, liver and intestines; on the one hand it should be noted that these tissues are normally not consumed and thus there would be no damage to human health [10], on the other hand, toxicological studies linking MPs with cell membranes, internalization and consequent cellular damages, are still preliminary.

Human routes of exposure to micro and nano plastics are different, but ingestion poses the greatest risks to humans, through NPPs-contaminated food. The in-vitro models have shown that NPs characteristics such as shape, charges and dimensions are very important for possible toxicity. The exposure of chicken intestinal cells to 50 nm diameter NPs has shown a reduction in the absorption of iron [11], which can be linked to the surface chemistry of the particles. However, possible local effects on the intestine are foreseeable, once the NPPs present in the lumen will interact with the complex fluid through adsorption reactions; in fact, local effects may occur in the intestine, as the MPs present in the lumen will certainly interact with its complex fluid through adsorption reactions facilitated by the size of their surface and by their charges [12]. The NPP-intestine interaction could affect the intestinal immune system and therefore cause local inflammation [13] which will consequently influence the absorption of the particles [14].

Ingestion of nanoparticles leads to a systemic condition only if the particles are absorbed through the intestinal barrier and distributed to the organs and / or tissues through the lymphatic and blood systems [15].

According to an EFSA provisional opinion, about 90% of ingested micro- and nano polystyrene particles are expelled with the feces [16] and 10% are absorbed by passive diffusion. In particular, the uncharged and smaller particles have a greater lipophilicity with a consequent better ability to overcome the intestinal membrane [17]. Cells are able to internalize foreign particles (phagocytosis), such as micro and NPPs, through processes of endocytosis or pinocytosis (NPs <150nm) [18, 19]. The internalized xenobiotic particles are able to activate the (cells of the) innate immunity system as a first response to exogenous substances [20, 21].

The toxicity of nanoparticles is linked to the formation of reactive oxygen species [22, 23]. It has been demonstrated that MPs and NPPs can induce ROS generation depending on different particle characteristics such as shape, sizes, surface-characteristics, doses and exposure times [24, 25]. Many studies analyze the correlations between ROS production and the exposure of human intestinal cells to MPs and NPPs [26–28]. Cells have a variety of defense mechanisms to ameliorate the harmful effects of ROS, such as antioxidant molecules and enzymes that play a role in detoxification (copper zinc (SOD1) and manganese (SOD2) superoxide dismutase, Catalase and Glutathione peroxidase (GPx1)). Elevated levels of SOD, Catalase and GPx1 were detected in Pocillopora damicornis exposed to microplastics [23]. The small size of NPs gives the same physicochemical characteristics such as a greater surface / mass ratio and different surface charge potential, conferring them the ability to directly cross the lipid membranes and allowing easier absorption of free radicals with consequent translocation between membranes.

Plastics are a potential risk to human health due to the different synthetic polymers that compose them such as polystyrene, that could represent a danger due to the possible presence, in its matrix, of the carcinogenic styrene monomer. The potential risks of polystyrene nanoparticles (PNPs) for humans are not yet clear, as are the studies that demonstrate cellular responses induced by polystyrene particles; particularly relevant are the ingested NPs, which, due to their small size, become part of the food chain more easily.

In this work, the potential toxicity of NPs, was evaluated through cytotoxicity and genotoxicity studies; the study was performed on in-vitro cellular model: HCT116, colorectal adenocarcinoma cell line. This cell line was chosen because it is commonly used in in-vitro studies, and intestinal cells seem to be the preferred absorption route for plastics; in particular it has been seen that the gastrointestinal tract is the area of the fish where most of the NPs are found.

Before performing the toxicity tests, the PNPs were characterized at microscopical level by Scanning Electron Microscopy (SEM), EDX spectroscopy (Energy Dispersive X-ray Analysis) and Fourier Transformed Infrared Spectroscopy (FTIR). The biological assays provide insight into the basal cytotoxicity and genotoxicity of PNPs. To measure the cytotoxicity, we used MTS-[3-(4,5-dimethylthiazol-2-yl)-5-(3-carboxymethoxyphenyl)-2-(4-sulfophenyl)-2H-tetrazolium] assay (MTS test) after cell exposure at different PNP concentrations. The genotoxicity of PNPs was measured using the Cytokinesis-Block Micronucleus cytome (CBMN cyt) assay after PNPs exposure. Finally, we evaluated ROS production induced by PNPs exposure and carried out analysis and quantification of detoxifying enzymes.

## Materials and methods

### Polystyrene nanoparticles

The polystyrene nanoparticles (PNPs), were in aqueous suspension (10% WT), sized (mean diameter) 100 nm, at the 1.05 gr/cm^3^ density. PNPs were purchased from Sigma Aldrich (catalogue No. 43302).

### Polystyrene nanoparticles characterization

The characterization of PNPs was performed at three different levels: at the morphological level by Scanning Electron Microscopy (SEM), at the compositional level by x-ray microanalysis system (EDS probe) and by Infrared spectroscopic characterization.

The study of the morphology and the elemental analysis of PNPs were carried out by scanning electron microscopy (Gemini Field Emission SEM 500, ZEISS, Milan, Italy) equipped with an x-ray microanalysis system (EDS Oxford Inca 250 x-act) at the Microscopy Center, University of L’Aquila.

First of all, 1 μL of sample was deposited on a sample holder (stub) and allowed to air dry; subsequently, the sample was ’sputtered’ (sample coated with a thin (5 nm) chromium film) using Sputter Quorum 150T ES to make it conductive for measurement purposes. The SEM analysis was performed at different magnifications and the morphological analysis of the particles was done simultaneously in order to obtain the elemental composition of the particles through EDS microanalysis. Finally, the PNPs was studied by Fourier Transformed Infrared Spectroscopy (FTIR) to determine molecular and chemical composition. The infrared analysis was carried out with a FTIR Bruker Vertex 70V equipped with a Platinum ATR accessory for reflectance measurements (OPUS 7.8 software package, Bruker Optics, Ettlingen, Germany).

Five spectra were acquired in reflection mode in the spectral range 4000–400 cm^−1^ (spectral resolution 4 cm^−1^, 64 scans). The database Bruker OPUS Spectrum Library was used to identify the spectra.

### Cell culture

In vitro studies were carried out on the human colorectal adenocarcinoma cell line HCT-116, purchased from American Tissue Type Collection.

HCT116 adenocarcinoma cells were cultured in Dulbecco’s modified eagle medium (DMEM) supplemented with 10% fetal bovine serum (FBS), 100 IU / ml penicillin / streptomycin, 2 mM L-glutamine and cultured in an incubator. HERAEUS (Hera 150 cell, Thermo Electron Corporation, Langenselbold, Germany) with a 5% CO2 atmosphere, at 37° C. Unless otherwise indicated, the cell culture media, trypsin and the reagents used were purchased from Euroclone SpA. The maintenance of the culture and the subsequent treatments with PNPs were carried out with a laminar flow biological hood under sterile conditions. Once sub-confluence (70–80%) was reached, the cells were detached using 0.25% trypsin / EDTA and seeded at 1-2x10,000 cells / cm^2^.

### Evaluation of cell viability by MTS assay

To assess viability of HCT116 cell line we carried out the MTS assay using a CellTiter Cell Proliferation Test Kit (Catalogue No. G3582 Promega, Madison, WI, USA). The experiment was performed according to the protocol provided by the manufacturer.

The effect of PNPs (size 100 nm) on cell proliferation was evaluated following exposure at different concentrations (400 μg/mL, 800 μg/mL, and 1200 μg/mL) and time (4, 24, and 48 h).

The HCT116 cells were seeded at 2000 cells / cm2 and after 24 h they were exposed to different concentrations of PNP at the established times in a humidified incubator in a controlled atmosphere (5% CO2, 80% humidity, 37° C). Each experimental condition represents a technical triplicate and data refer to the mean and standard error of three independent experiments. Triton X-100 (0.1% concentration) was used as a positive controls for each series of experiments (4, 24, and 48 h). Cell culture absorbance was measured at 490 nm, and cell proliferation was evaluated [29].

### ROS (Reactive Oxygen Species) detection

The cellular ROS concentration was detected following the "Total ROS Assay Kit 520 nm" protocol (Catalogue No. MAK 143, Sigma-Aldrich Srl, Milan, Italy). Shortly, were seeded 10.000 cells/cm^2^ in 96-well plates, after 24 h, cells were incubated at 37°C for 1 hour with ROS stain (Catalogue No. MAK 143A) resuspended in dimethyl sulfoxide (DMSO). After incubation, the medium was removed and replaced with freshly DMEM in the control cells and DMEM with PNPs (at 100 μg/mL, 200 μg/mL, 400 μg/mL, 800 μg/mL and 1200 μg/mL concentrations) in treated cells. Regarding the positive control, H_2_O_2_ (hydrogen peroxide) was added at 150 μM concentration. The plate was read at different times in a microplate reader (Perkin-Elmer Victor 3) (λexc 490, λemi 535) at T0, T15, T30, T45 and T60 min and the fluorescence data were evaluated for statistical analysis. Each experimental condition represents a technical triplicate and biological duplicate; data refer to the mean and standard error.

### Western Blot analysis of superoxide dismutase 1 and 2, Catalase, Glutathione peroxidase (SOD1, SOD2, Catalase, GPx1)

Control and treated cells were harvested and lysed (10 × 10^6^ cells/ml) in RIPA buffer (cat. 9806; Cell Signaling Techonology) supplemented with protease inhibitor cocktail 100X (cat. 5871; Cell Signaling Techonology) and 1mM PMSF (CAT. 8553; Cell Signaling Techonology).

After centrifugation at 8,000 × g for 60 min at 4°C, supernatants were assayed for protein content, by using the Pierce BCA Protein Assay Kit and bovine serum albumin as the standard (cat. 23227; Thermofisher Scientific). Each sample (25 μg) added with the sample buffer 1:1, was run on polyacrylamide gels (12%), according to *Laemmli* [30]. Proteins were transferred onto polyvinylidene difluoride (PVDF) sheets by electrophoretic transfer [31]. Non-specific binding sites were blocked at room temperature for 1 hour with 5% (w/v) fatty-acid free milk, in Tris-buffer saline containing 0.05% (v/v) Tween-20 (cat. P5927; Sigma-Aldrich) (TBS-T). Membranes were incubated overnight with 1 μg/mL of primary antibodies diluted in TBS-T, and then with the peroxidase-conjugated secondary antibody for 2 h. Primary antibodies (SOD1, SOD2, Catalase, Actine and GPx1) and secondary antibodies (Anti-Rabbit IgG HRP-linked and Anti-mouse IgG HRP-linked) were purchased from Cell Signaling Technology.

Protein bands were visualized using ECL WEST pico PLUS substrate detection system kit (cat. ECL-2001 Immunological Science, Roma, Italy) and the signal was aquired by Alliace^TM^ Q9-Series (UVItec Limited, UK. Cowley Road, Cambridge, CB4OWS, United Kingdom) and ImageJ (U. S. National Institutes of Health, Bethesda, Maryland, USA). β-actin was used as the loading control for data normalization. Results were given as arbitrary units. Three independent experiments were permorfed.

### Cytokinesis-Block Micronucleus (CBMN cyt) cytome assay

CBMN assay was performed according to the *Fenech* [32] protocol and *OECD guidelines* [33]. The HCT-116 cell line was seeded in each flask with 1.5 × 10^5^ cells/flask and after 24 h the cells were exposed to different concentrations of PNPs (800 μg/mL, 1200 μg/mL and Colchicine was used as a positive control at 5 μg/mL concentrations) for 48 h. Then, Cytochalasin B (3 μg/mL) was added to the cell cultures and after 24h the cells were detached. Cells were harvested and centrifuged for 10 min at 1000 rpm.

Three flasks for each condition were detached and the cells mixed to obtain a single pellet. Each pellet (approximately 10 x 10^6^ cells/ml) was harvested with PBS, prefixed with methanol/glacial acetic acid (3:5) and spread onto three glass slides (20 μl of cell suspension per slide). After air-drying, the cells were fixed with methanol/glacial acetic acid (6:1) for 10 min and stained with 5% Giemsa solution for 8 min. All procedures were conducted at room temperature. After washing with distilled water, the slides were rapidly dried in xylene and mounted with Canadian balsam. By using a Leitz light microscope, cells were analyzed for each treatment following the *Fenech* guidelines [32].

For each experimental condition, we calculated Micronuclei frequency (MN) and the Cytokinesis Block Proliferation Index (CBPI) to determine the frequency of mononuclear, binucleated and multinucleated cells, using the formula: [(N° mononucleated cells) + (2 × N° binucleated cells) + (3 × N° multinucleated cells)]/ (total number of cells). Moreover, for each experimental condition we evaluated Nuclear Buds (NBUDs) as a biomarker of genotoxicity.

### Statistical analysis

Statistical analysis was performed using GraphPad Prism software, version 6.0 (© 1995–2015 GraphPad Software, Inc. San Diego, CA 92108). Three independent experiments were conducted for all tests. The data were analyzed by Student’s t test (unpaired) with post-hoc correction, comparing the value of the treated cells with the respective untreated control, through independent tests. For statistically significant values, we assume * = *p <0.05*; ** = *p <0.005*; *** = *p <0.0005*.

## Results

### PNPs characterization

**Fig 1:**
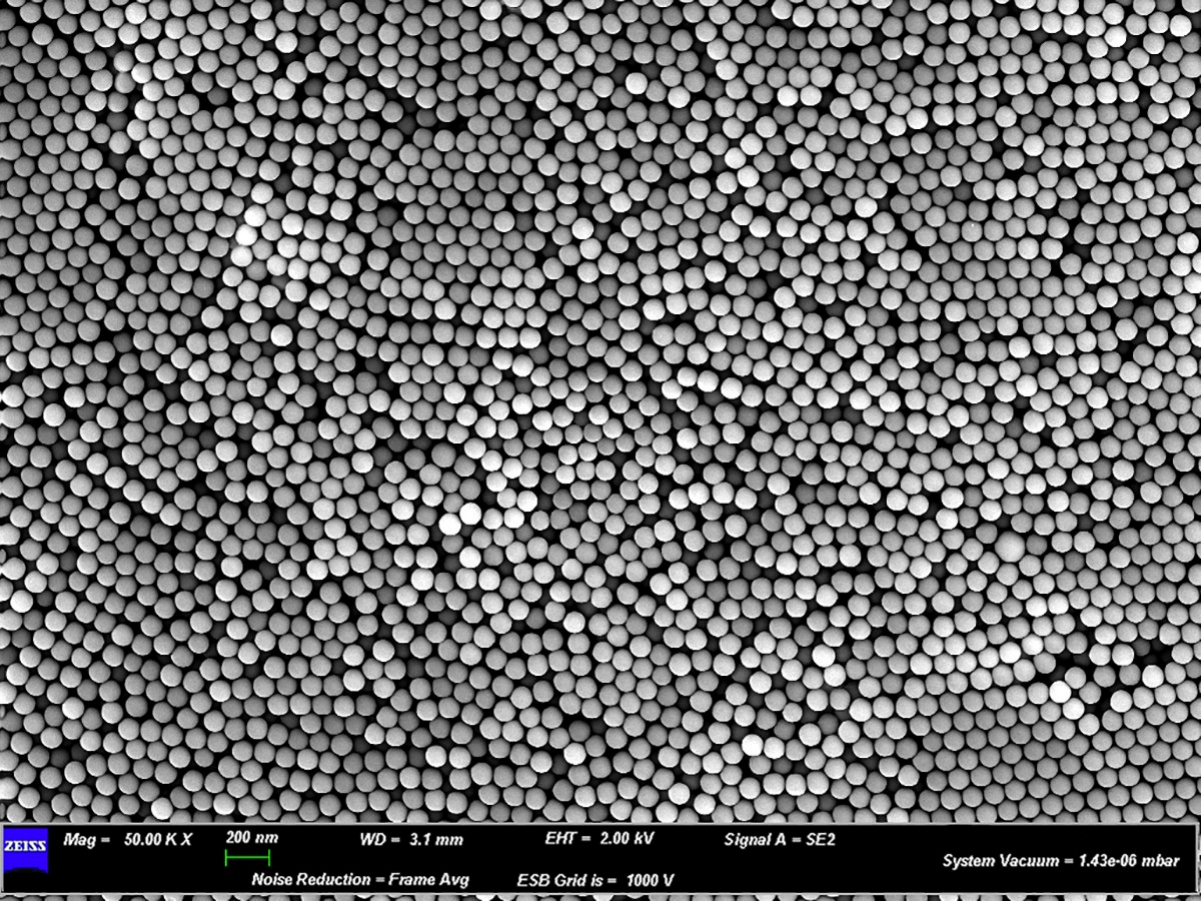
Scanning electron microscopy (SEM). Polystyrene Nanoparticles morphological analysis with SEM.

Thanks to the morphological analysis, by SEM microscopy (Fig 1), that investigate about size and dimension of PNPs, it is possible to note that the nanoparticles are homogeneous and with the same size (100nm). As shown in Fig 1, the polystyrene nanoparticles tend to form a homogeneous tridimensional structure sometimes clumping together due to their physicochemical properties (shape, surface charge, size).

**Fig 2:**
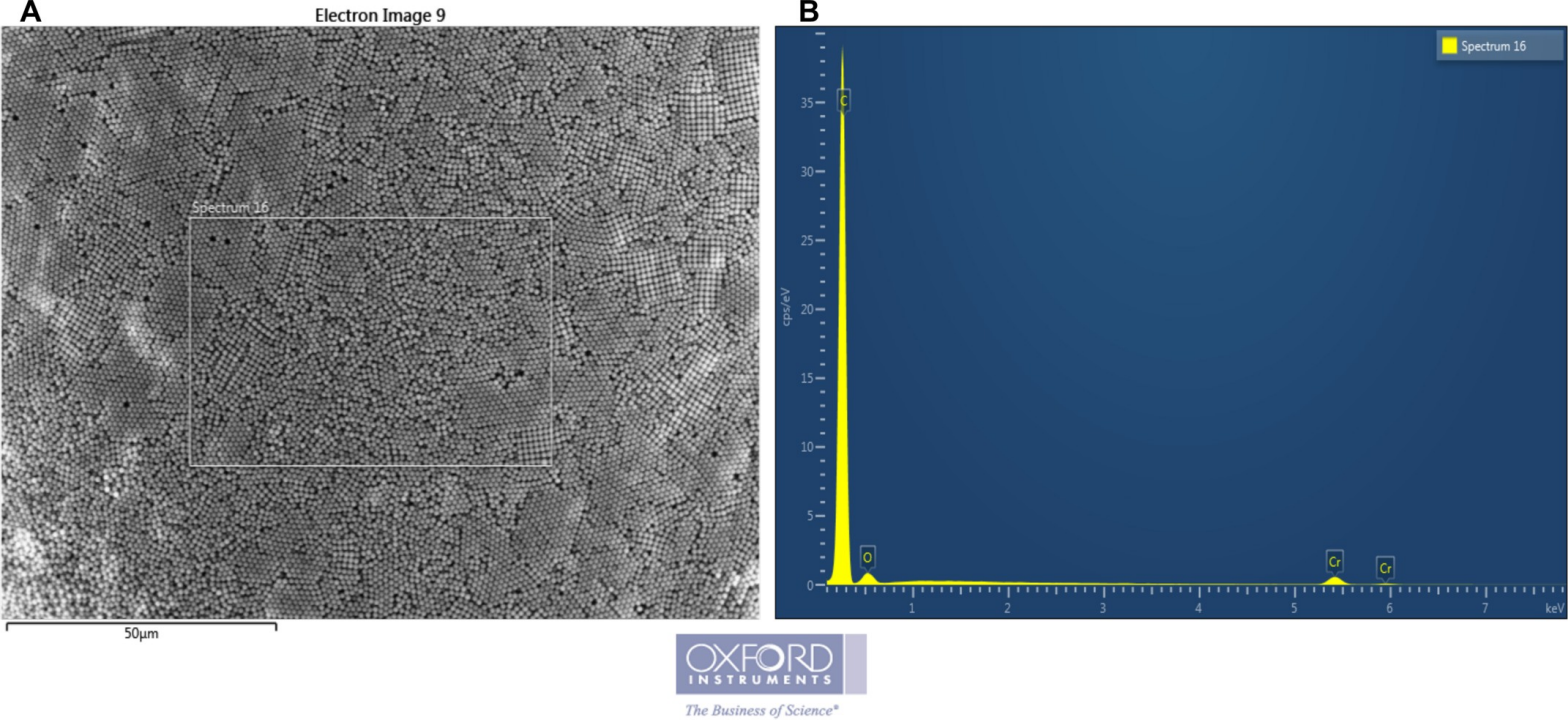
Energy dispersive x-ray analysis (EDX) of Polystyrene nanoparticles. Area of sample (a) and relative Elemental Spectrum (b).

Regarding the composition, the x-ray microanalysis system (EDS probe) shows in Fig 2 the elemental composition of PNPs, to assess and confirm their inorganic composition. Fig 2A shows the area of interest for which the analysis of the inorganic elements was carried out. Fig 2B shows the relative spectrum of the selected area; the results of the PNPs sample spectrum show the presence of, Carbon, Oxygen and Chromium, the latter present in traces due to the Sputtering process in the sample preparation.

Thanks to the microanalysis it was possible to confirm the manufacturer’s specifications, but above all that there are no elements other than the polystyrene component (i.e., heavy metals), which can affect and negatively influence the consequent toxicity *in-vitro* tests.

**Fig 3:**
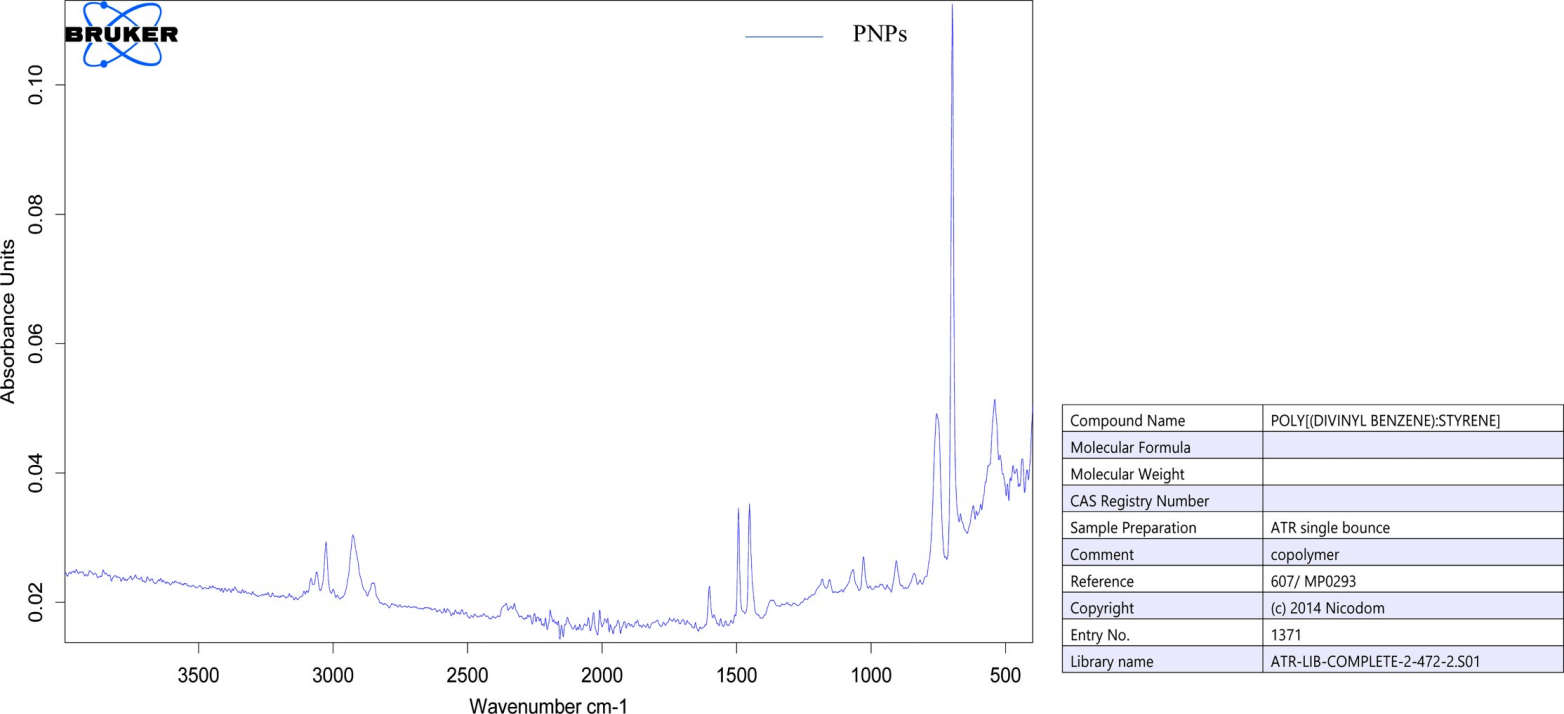
The Fourier Transformed Infrared Spectroscopy (FTIR) of polystyrene nanoparticles. Sample Spectrum and relative characteristics table.

Finally, for a complete microscopic characterization and in order to confirm electron microscopy results, Fig 3 shows the Fourier Transformed Infrared Spectroscopy (FTIR) to determine molecular and chemical composition.

Through this technique it is possible to obtain a very fine characterization; in fact the spectrum obtained (Fig 3) confirmed that PNPs are composed of Poly[(divinyl-benzene)Styrene], the table on the side shows all the characteristics of the sample and related references of the Bruker OPUS Spectrum Library thanks to which it was possible to identify the sample of interest.

### MTS assay

Regarding the PNP concentrations used, in the literature it has been calculated that the total number of plastic particles ingested per person per year through fish products and drinking water consumption is approximately 11.000 and 10.220 respectively [34–36].

Therefore, as regards the measurement of cell viability after exposure (4, 24, and 48 h) to PNPs, we used three different concentrations: 400 μg/mL, 800 μg/mL, and 1200 μg/mL.

**Fig 4:**
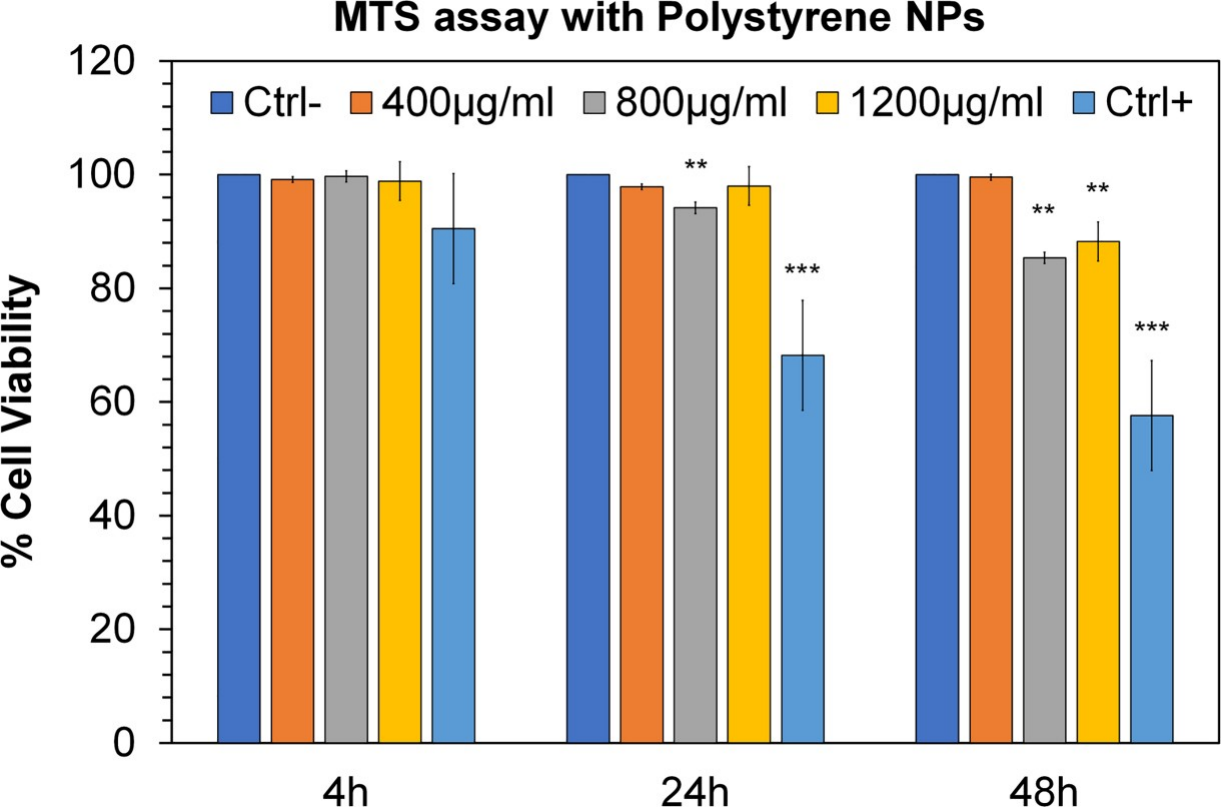
MTS test in HCT116 cells. The effects of polystyrene nanoparticles (PNPs) on HCT116 cell viability after exposures at 4, 24, and 48 h at 400, 800, and 1200 μg/mL concentrations compared to the control cells. Triton-X-100 used as a positive control. Significance values * ** = *p* < 0.005; *** = *p* < 0.0005; error bars represent the standard error of the mean.

Through the MTS assay we evaluated cell viability after exposure to PNPs, which, as reported in the Fig 4, does not show statistically significant changes within 4 hours. Subsequently, at 24h we can see significant changes only for the condition of 800 μg / mL, where there is a reduction in cell viability of about 6% compared to the control. These data were confirmed at 48 h, where the concentration of 800μg / mL induces a marked reduction in viability, of about 15%, followed by the concentration of 1200μg / mL which in turn shows a reduction in viability of about 12%.

### ROS detection

**Fig 5:**
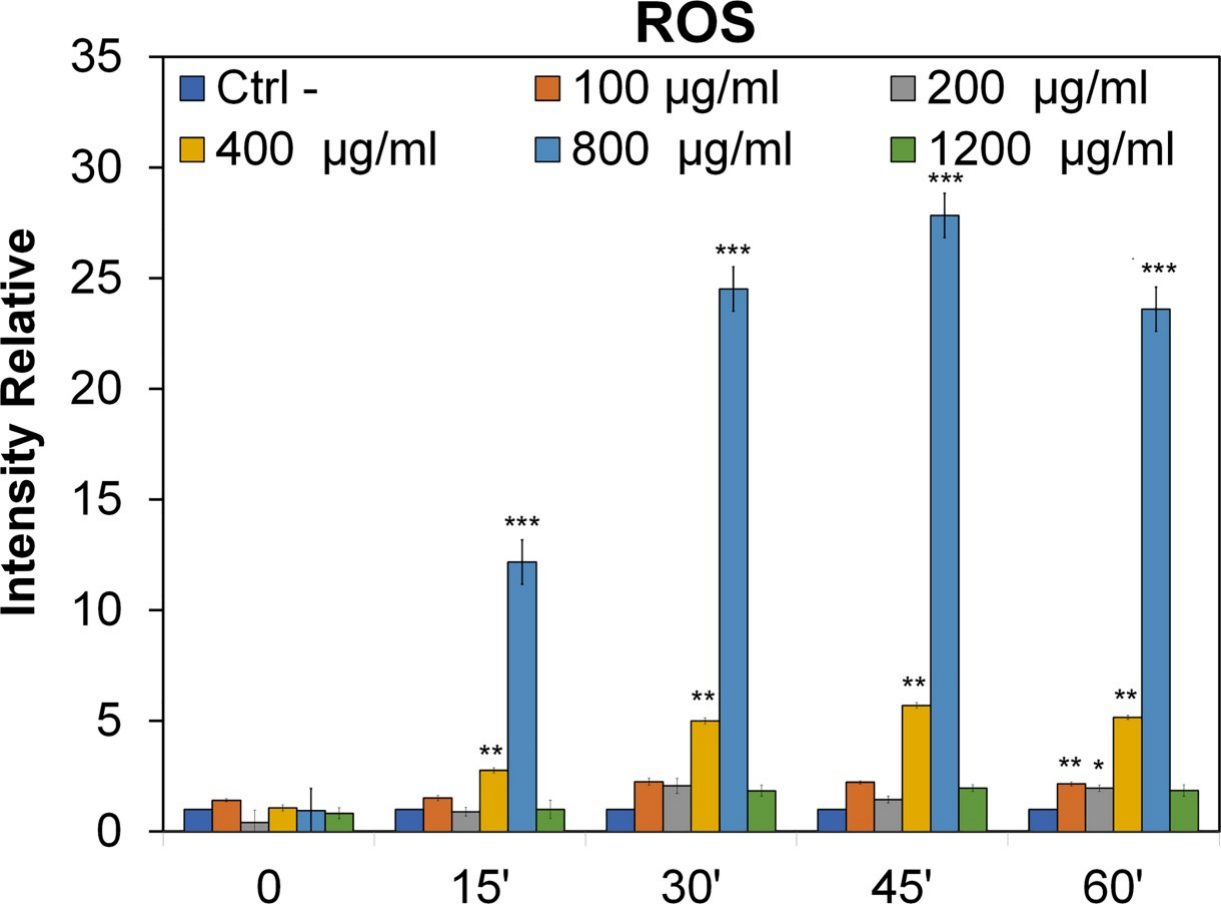
ROS production in HCT116 cell line. The ROS production after exposure at different concentrations and time (100 μg/mL, 200 μg/ml, 400 μg/mL, 800 μg/mL and 1200μg/mL concentrations and at 0,15,30,45 and 60 min time) of Polystyrene Nanoparticles on HCT116 cells. Each time refers to T0. Hydrogen peroxide (H_2_O_2_) was used as a positive control (value not reported in the Figure). Significant values * = p < 0.05; ** = p < 0.005; ***p = <0.0005; error bars represent the standard error of the mean.

The probable production of reactive oxygen species (ROS) in the presence of PNPs, at different concentrations (100 μg / mL, 200 μg / mL, 400 μg / mL, 800 μg / mL and 1200 μg / mL) was determined as a function of the time course of the experiments. As shown in Fig 5, a significative ROS increase was produced at concentrations of 400 and 800 μg / mL. Specifically, at 15 min the concentration of 400 μg / mL triples the production of ROS compared to the control, followed by the concentration of 800 μg / mL where there is a significant increase in production, rising from a value of 1 of relative intensity (control), up to a value of 12 times higher. The same situation is confirmed after 30 min, in this case we found relative intensity values from about 5 at 400 μg / mL to about 25 at 800 μg / mL where the production seems to have doubled compared to 15 min.

It is after 45 minutes that the maximum production of ROS is detected, in fact the relative intensity at concentrations of 400 μg and 800 μg increases until it reaches values of about 6 and 28 respectively. After 1 h treatment the increase in ROS production can be observed at all concentrations except 1200 μg / mL. In particular, however, the values (compared to the other times) begin to drop at 400 μg and 800 μg, but at the same time a slight production of ROS begins at 100 and 200 μg / mL. The lower concentrations of PNP (100 and 200 μg / mL), which produce ROS in longer exposure times, are able to activate the cells giving a delayed response that could mimic the chronic effect of exposure.

### Micronuclei test with block of cytokinesis by Cytochalasin B (CBMN assay)

**Fig 6:**
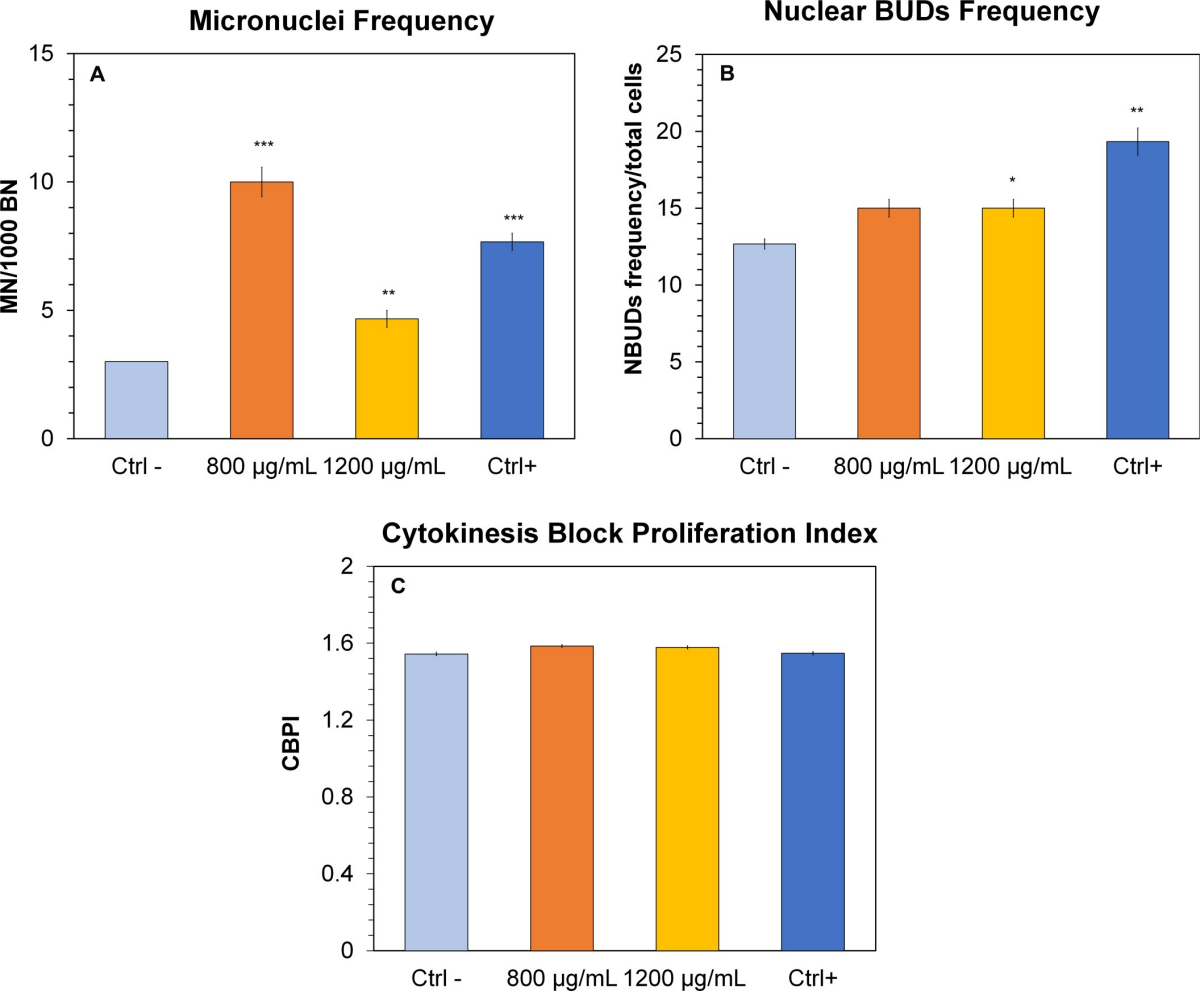
Micronuclei (BMN), Nuclear Bud (NBUDs) and Cytokinesis Block Proliferation Index (CBPI) in the HCT116 cells treated with polystyrene nanoparticles (PNPs). (**a**) Micronuclei: the number of MN refers to 1000 binucleated cells, (**b**) Nuclear Buds: refers to a total of 1000 binucleated cells and (**c**) CPBI, were evaluated at 800, 1200 μg / mL after 48h exposure to PNPs. CBPI = ((N° mononucleated cells) + (2 x N° binucleated cells) + (3 x N° multinucleated cells))/ (total cell number). Colchicine was used as a positive control. Significance values * = p < 0.05; ** = p < 0.005; *** p = 0.0005; error bars represent the standard error of the mean.

Fig 6 shows the data obtained by analyzing the genotoxic potential of PNPs, expressed as frequency of micronucleus formation (Fig 6A); in particular, at the concentration of 800 μg / mL the number of micronuclei is tripled compared to the control, while at 1200 μg / mL the micronuclei switch from a value of 3 (Control) to a value of about 5. This is according to the viability test (MTS test), in fact at 48 h (same exposure time of the micronuclei) the cell viability at concentrations of 800 and 1200 μg / mL was significantly reduced. Furthermore, in correlation with the micronucleus analysis, we report the formation of Nuclear Buds (Fig 6B), protrusions of the nucleoplasm, also an index of genotoxic damage: in this case we found a statistically significant increase in the formation of NBUDs at the concentration of 1200 μg / mL.

**Fig 7:**
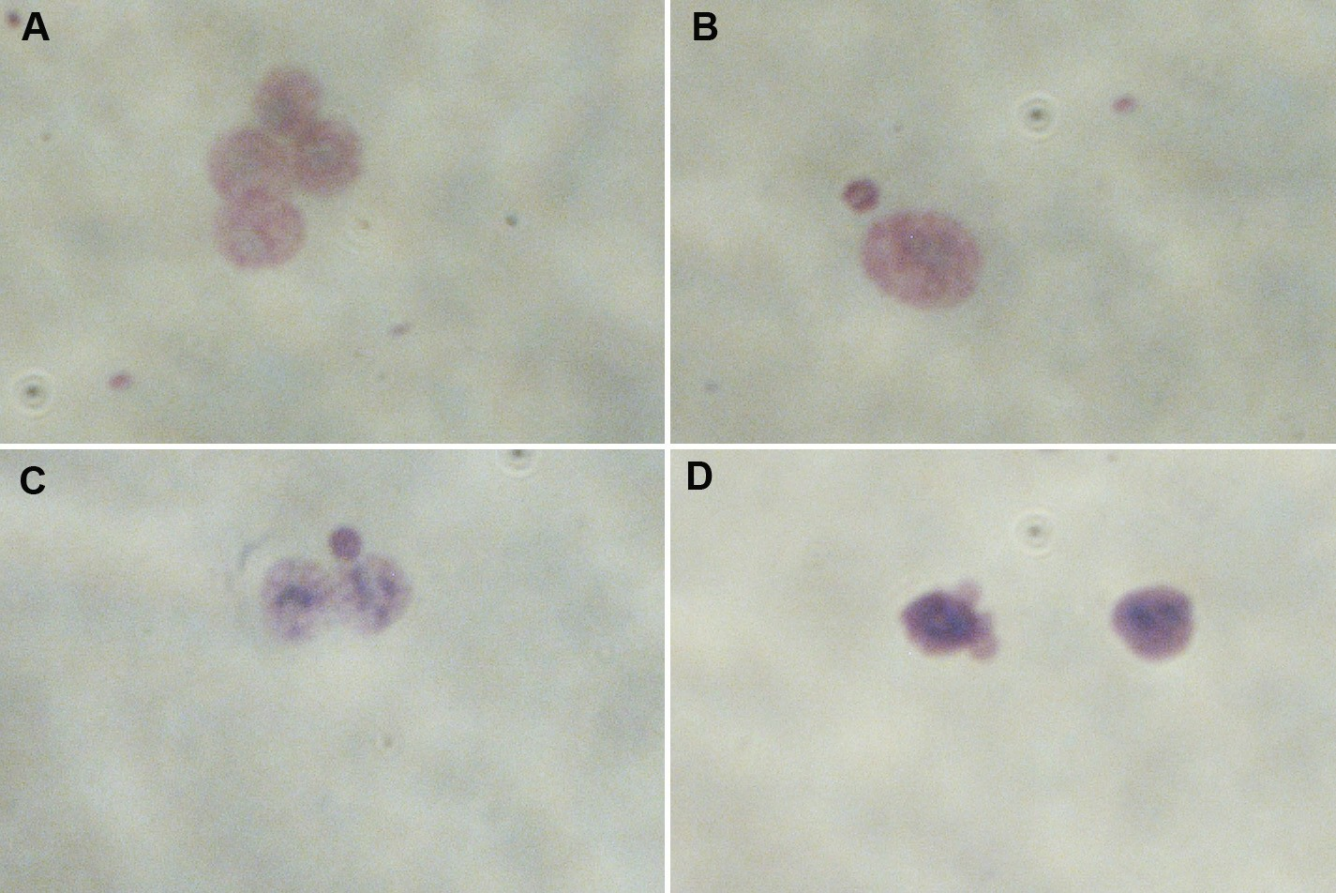
Cytokinesis-Block Micronucleus cytome (CBMN cyt) assay in HCT116 cells. DNA damage after PNPs exposure, **(A)** Control cells, **(B)** mononucleated cell with MN, **(C)** binucleated cell with MN and **(D)** nuclear blebs. Magnification 40X.

Finally, we evaluated the Cytokinesis Block Proliferation Index (CBPI) in order to determine the frequency of mono-, bi- and multinuclear cells as well as the progression of cell proliferation and consequent cytostatic effect given by treatment with PNPs. As shown in Fig 6C, the CBPI analysis does not show any statistically significant differences. In Fig 7, we can see control cell (A) and DNA damage as micronuclei (B and C) in HCT116 cells after PNPs exposure.

### Western Blot analysis of superoxide dismutase 1 and 2, catalase, glutathione peroxidase

**Fig 8:**
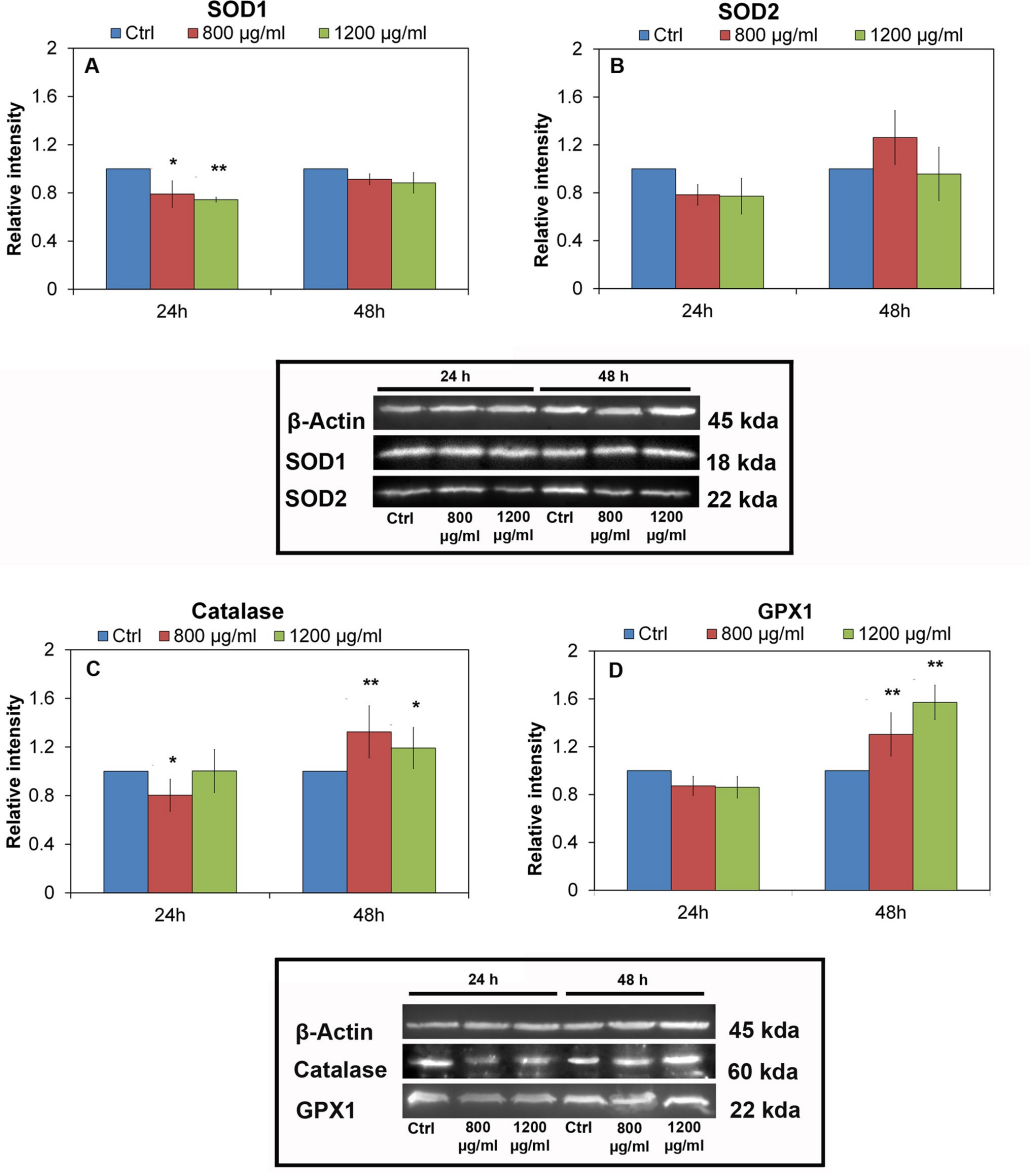
Western Blot analysis. Superoxide dismutase 1(**a**) and 2(**b**) (SOD1-SOD2) respectively immunoblotting, and Catalase (**c**) and Glutathione peroxidase (GPx1) (**d**) respectively, of extract of HCT-116 cell line in presence of PNPs: 800 μg / mL and 1200 μg / mL PNPs concentrations for 24 and 48h, histograms normalized vs ß-actin, Significant values * = p < 0.05; ** = p < 0.005; ***p = <0.0005; error bars represent the standard error of the mean.

Regarding the determination of the protein pattern involved in the cell detoxification process, we analyzed SOD1-SOD2 (free radical detoxification, Fig 8A and 8B) and Catalase-GPx1 (superoxide anion detoxification, Fig 8C and 8D). As for SOD1, a statistically significant reduction at 800 μg / mL and 1200 μg / mL can be observed only at 24h; while an increase at 48h is observed for SOD2, of about 25% at the concentration of 800 μg / mL and there are no significant variations at 1200 μg / mL. The behavior of SOD2 is justified by the fact that it is activated in order to subtract peroxides, transforming superoxide anion into hydrogen peroxide and molecular oxygen.

In the case of catalase after 24 hours, a slight but significant decrease in enzymatic expression is observed at 800 μg / mL. On the contrary, at 48 h from the treatment, there is an increase of 30% and 20% to 800 μg / mL and 1200 μg / mL, respectively. Glutathione peroxidase does not undergo expression variation at 24 h while it increases at 48 h at both 800 μg / mL and 1200 μg / mL, specifically by 30% and 57% respectively. The ß-actin used as housekeeping protein (loading control) shows constant expression under all conditions.

## Discussion

Plastics are now ubiquitous: in the environment, 5.25 trillion plastic particles have been estimated to be dispersed in the oceans alone, representing a danger to living organisms, including man [37–39].

The danger is determined by several factors: ingestion of plastic objects and subsequent suffocation and intestinal obstruction, absorption of micro- and nanoplastics [12, 40, 41].

Plastic objects in fact undergo fragmentation causing the release and formation of micro- and nanoparticles that are absorbed in particular at the level of the digestive systems of the organisms that take them, in fact the main route of ingestion is the oral one through food [42–44].

Exposing human colorectal carcinoma cells (HCT116) to NPs, no alterations in cell viability (MTS assay) were observed except at high doses. The choice of high concentrations was also dictated by the desire to determine the concentrations with the greatest toxic effect. With the new emerging pollutants (such as polystyrene) it is important to understand when the negative consequences generated by the toxicity of the PNPs themselves begin.

Considering that, to date, we cannot estimate an average for the environmental concentration of nanoplastics in the globe, and that it is still difficult to fully understand the accumulation process in the human body, it is important to include all the concentrations that lead to potential toxic effects. Therefore, starting from the concentrations reported in the scientific literature that did not lead to any significant effects in terms of toxicity, we increased them in order to verify their actual toxicity.

Regarding MTS assay, only at concentrations of 800 μg / mL and 1200 μg / mL there were relative percentage decreases in cell viability, compared to the untreated control. As reported in the literature, cytotoxicity could be associated with an increase in PNPs accumulation at the intracellular level [45, 46]; in addition, the SEM-EDX analysis of PNPs highlights the presence exclusively of C and O, while FTIR analysis confirms their expected chemical nature (Poly[(divinyl-benzene)Styrene]), therefore the cytotoxic potential could depend mainly on the physicochemical properties (charge, shape, size) [47–49]. Furthermore, it has been shown that properties and dimensions of nanoparticles greatly influence the permeability of these through membranes and intestinal mucus, for example NPs with a neutral surface are able to more easily cross intestinal barriers than particles with different surface properties [49].

The cause of the increased mortality could also refer to an increase in oxidative stress. In this regard, as a marker for the evaluation of the mechanisms involved, we measured the concentrations of ROS that can cause severe oxidative damage, especially to DNA, lipids and proteins. There are numerous metabolic activities that lead to the production of ROS: smoking, intense physical exercise, unbalanced diets, sunlight, alcohol, pollution are all causes of overproduction. Since the presence of ROS is ’normal’, organisms have developed natural antioxidant systems that can be divided into enzymatic and non-enzymatic, consequently we evaluated the variation of enzymes such as SOD1 and 2, Catalase and Glutathione peroxidase.

The correlation between the size of the particles and the potential to generate ROS (based on their physicochemical characteristics) is significant, the smaller the particles, the greater their internalization and consequently the ROS generation [25, 50, 51]. The damage caused by ROS can be found at a physiological, cellular, genetic level, up to tumorigenesis processes [52].

Indeed, PNPs are able to induce increased ROS production. This was observed as early as 45 minutes after treatment at a concentration of 800 μg / mL. Lower doses also induced stress, albeit less, after a longer exposure time (1h), probably not enough to affect cell growth. The higher dose of NPs, 1200 μg / mL, on the other hand, has a lower effect than the other ones. Probably this is due to aggregation phenomena of the nanoparticles at high concentrations or to molecular mechanisms other than oxidative stress [53]. It is also necessary to consider that the cells used are tumor cells with accelerated replication and transcription mechanisms. Changes in ROS have been observed in healthy cells at concentrations as low as 5 μg / mL [54]. Several studies have found that high ROS production by NPs could cause not only oxidative damage, but also possibly induce apoptosis and necrosis [55, 56]. Massive ROS production was observed *in vitro* on human cells after exposure to various types of NPs [57, 58].

Oxidative stress by PNPs was also observed in *in-vivo* organisms, in particular in the *Zebrafish* model and in bivalve spermatozoa. In both models’ defects and difficulties in movement were found, in *Zebrafish* a difficulty in obtaining food was observed, while in bivalve spermatozoa lower fertility and lower ability to fertilize egg cells were reported [59–61].

The production of reactive oxygen species causes an increase in the synthesis of enzymes responsible for cell detoxification. Since protein synthesis is a slow process, the above variation was found 48 hours after exposure to PNPs. In particular, SOD2, catalase and glutathione peroxidase participate in the detoxification process; SOD1 did not increase relative to the negative control indicating that it is probably not involved in this specific process.

NPs in general induce genotoxicity and possibly carcinogenesis, in fact there are several studies reported in the literature, which show how a prolonged inflammatory state together with oxidative stress, can directly damage DNA. The inflammatory cascade and ROS production can both cause mutagenesis, due to the oxidation / hydrolysis of nucleic acids, and trigger the tumorigenic process due to mutations such as translocations or deletions etc.

It has been shown that NPs are able to enter the cell nucleus and therefore have a direct interaction with DNA, causing physical damage to the genetic material [62]. Regarding genotoxic damage, our data show a MN frequency, at the concentration of 800 μg / mL, tripled compared to the control, followed by the concentration of 1200 μg / mL. The damage to the genetic material can follow indirect mechanisms where there is no direct contact with the DNA, or through interaction with the proteins involved in the replication and / or repair of DNA damage. Furthermore, the nanoparticles can activate numerous cellular responses that induce genotoxicity, such as the production of reactive oxygen species, inflammation, incorrect cell signaling [63], although in the literature, the main cause of cyto-genotoxicity from NPs, is given from the production of reactive oxygen species [63].

## Conclusion

Considering the results obtained in this work, it is possible to hypothesize that at high doses of PNPs the cell line HCT116 undergoes significant oxidative stress (already at the earlier exposure times) with a consequent reduction in cell viability (after longer exposure and high concentrations) and an increase in the biosynthesis of detoxifying enzymes. The concentration that causes the most significant reduction in viability, probably due to high ROS production, is 800 μg / mL; these data have been confirmed by the CBMN assay, where the nuclear damage is more significantly detectable at the concentration of 800 μg / mL both with formation of MN and NBUDs. The genotoxicity tests could be used to understand if clastogenic effects are present following exposure to PNPs.

Several studies have suggested that ingesting plastic particles through food and / or drinking water may be a sufficient source for the absorption of plastic nanoparticles. Through our study we have highlighted that direct contact of PNPs (in the culture medium) with cells can potentially cause health problems through the production of ROS and DNA damage.

There are many other types of plastics, in many different concentrations, that deserve attention and consequent analysis, as well as polystyrene itself which needs further investigation both through *in-vitro* studies and with *in-vivo* models (e.g., *Drosophila*)

In conclusion, the effects of nanoplastics are mainly chronic and therefore long-term, and to date little is known about them, but all the *in- vitro* and *in vivo* studies show how by increasing concentrations, inflammatory, cytotoxic and genotoxic phenomena increase; similar concentrations can be reached with the bioaccumulation process. It is therefore essential to increase environmental and human toxicology studies to understand the cumulative effects of exposure to nanoplastics and to extend application studies to try to understand how to solve the pollution problem derived from them.

## Supporting information

S1 File Data MTS. MTS test in HCT116 cells treated with Polystyrene Nanoparticles.
(PDF)

S2 File Data ROS. ROS detection in HCT116 cells treated with Polystyrene Nanoparticles.
(PDF)

S3 File Data CBMN. CBMN test in HCT116 cells treated with Polystyrene Nanoparticles.
(PDF)

S4 File Data Western Blot analysis. Western Blot on the HCT116 cells treated with Polystyrene Nanoparticles.
(PDF)

S1 Raw images Reporting Western Blot gel data analysis. Western Blot, SOD1 and 2 analysis. Western Blot Catalase and GPx1 analysis.
(TIF)
