# Supplementary material for: Genotoxicity and oxidative stress induction by polystyrene nanoparticles in the colorectal cancer cell line HCT116
Source: PLoS One. 2021 Jul 23;16(7):e0255120. doi: 10.1371/journal.pone.0255120 (PMC8301662; doi:10.1371/journal.pone.0255120)
Supplement: S1 File — MTS test in HCT116 cells treated with Polystyrene Nanoparticles. (PDF) [file pone.0255120.s001.pdf]

MTS1

4H

C-	400	800	1200	C+
1,2647	1,218	1,2555	1,3115	1,121
1,2818	1,2242	1,2464	1,2964	1,0191
1,3045	1,2869	1,3153	1,2776	0,9932
1,162	1,2898	1,268	1,2544	1,0956
1,2561	1,215	1,2275	1,2552	1,0636

Mean 1,25382

Mean %

C-	400	800	1200	C+
100	97,1431	100,13	104,6	89,4068
100	97,6376	99,408	103,396	81,2796
100	102,638	104,9	101,897	79,2139
100	102,87	101,13	100,046	87,381
100	96,9039	97,901	100,11	84,8288

24H

1,2729	1,2915	1,2432	1,2726	0,8256
1,2531	1,1922	1,1978	1,2304	0,8915
1,2934	1,074	1,2119	1,2247	0,8481
1,2385	1,216	1,1281	1,2429	0,7806
1,2151	1,2317	1,1222	1,2139	0,7671
1,2235	1,156	1,2444	1,2708	0,7882

Mean 1,24942

100	103,368	99,502	101,856	66,0788
100	95,4205	95,869	98,478	71,3533
100	85,9601	96,997	98,0217	67,8797
100	97,3254	90,29	99,4784	62,4772
100	98,582	89,818	97,1573	61,3966
100	92,5232	99,598	101,711	63,0854

48 H

1,7351	1,8299	1,389	1,3914	0,8606
1,6638	1,7217	1,3515	1,3117	0,8588
1,5599	1,7043	1,1266	1,3301	0,8374
1,6252	1,6401	1,147	1,3316	0,756
1,6329	1,698	1,196	1,3185	0,8617
1,6119	1,6718	1,3575	1,351	0,8847

Mean 1,63813

100	111,706	84,792	84,9381	52,5354
100	105,101	82,502	80,0728	52,4255
100	104,039	68,773	81,1961	51,1192
100	100,12	70,019	81,2876	46,1501
100	103,655	73,01	80,488	52,6026
100	102,055	82,869	82,4719	54,0066

MTS2

4H

C-	400	800	1200	C+
1,3895	1,3548	1,3436	1,3979	1,3499
1,5037	1,3724	1,3915	1,3414	1,4564
1,392	1,4221	1,3861	1,4059	1,3299
1,5324	1,4761	1,4635	1,4293	1,391
1,5022	1,5088	1,4477	1,4205	1,4681
1,5167	1,4763	1,4871	1,4944	1,5619

Mean 1,47275

Mean %

C-	400	800	1200	C+
100	93,1862	94,483	91,0813	98,8898
100	96,5609	94,116	95,4609	90,3005
100	100,227	99,372	97,0497	94,4492
100	102,448	98,299	96,4522	99,6843
100	100,241	100,97	101,47	106,053

24H

1,2917	1,3034	1,2802	1,252	0,8327		100	99,0752	97,312	95,1681	63,2959
1,2859	1,2887	1,2073	1,1675	0,8328		100	97,9578	91,77	88,745	63,3035
1,2843	1,2557	1,2785	1,3228	0,8491		100	95,4494	97,182	100,55	64,5425
1,2859	1,3721	1,2212	1,2537	0,8427		100	104,297	92,827	95,2973	64,056
1,3596	1,3227	1,2982	1,3176	0,8172		100	100,542	98,68	100,155	62,1177
1,386	1,3604	1,3486	1,3447	0,8008		100	103,408	102,51	102,215	60,8711

Mean 1,31557

48H

1,2549	1,2304	1,2157	1,2317	0,878		100	93,218	92,104	93,3165	66,5194
1,2115	1,1849	1,2028	1,242	0,8653		100	89,7708	91,127	94,0968	65,5572
1,345	1,2573	1,2051	1,246	0,8686		100	95,256	91,301	94,3999	65,8072
1,3458	1,2657	1,2686	1,2145	0,7742		100	95,8924	96,112	92,0134	58,6552
1,4256	1,2568	1,2322	1,2844	0,7819		100	95,2181	93,354	97,3092	59,2386
1,3367	1,2995	1,2969	1,2832	0,8839		100	98,4532	98,256	97,2183	66,9663

Mean 1,3199

MTS3

4H

C-	400	800	1200	C+	Mean % C-	100	400	800	1200	C+
1,1238	1,1085	1,1217	1,1061	0,9424		100	101,836	103,05	101,615	86,5765
1,072	1,0768	1,1133	1,0981	0,8902		100	98,9236	102,28	100,88	81,781
1,0858	1,0928	1,1237	1,0275	0,9125		100	100,394	103,23	94,3945	83,8297
1,0828	1,0928	1,0714	1,0793	0,9774		100	100,394	98,428	99,1533	89,7919
1,1082	1,1044	1,1428	1,0848	1,0628		100	101,459	104,99	99,6586	97,6375
1,0585	1,093	1,097	1,0693	1,0389		100	100,412	100,78	98,2346	95,4418

Mean 1,08852

24H

1,3746	1,3576	1,2197	1,3709	1,1454		100	101,087	90,819	102,077	85,2867
1,3342	1,2199	1,1748	1,2566	0,9502		100	90,834	87,476	93,5666	70,752
1,3511	1,3133	1,2324	1,3515	1,0845		100	97,7885	91,765	100,633	80,752
1,2895	1,336	1,2549	1,2324	0,8059		100	99,4788	93,44	91,7647	60,0074
1,3772	1,3202	1,21	1,2975	1,1186		100	98,3023	90,097	96,6121	83,2911
1,3314	1,3457	1,1946	1,3477	1,0317		100	100,201	88,95	100,35	76,8206

Mean 1,343

	4H					24H				
	100	97,143	100,13	104,6	89,407	100	103,368	99,502	101,856	66,0788
	100	97,638	99,408	103,4	81,28	100	95,4205	95,869	98,478	71,3533
	100	102,64	104,9	101,9	79,214	100	85,9601	96,997	98,0217	67,8797
	100	102,87	101,13	100,05	87,381	100	97,3254	90,29	99,4784	62,4772
	100	96,904	97,901	100,11	84,829	100	98,582	89,818	97,1573	61,3966
	100	91,991	91,231	94,918	91,658	100	92,5232	99,598	101,711	63,0854
	100	93,186	94,483	91,081	98,89	100	99,0752	97,312	95,1681	63,2959
	100	96,561	94,116	95,461	90,3	100	97,9578	91,77	88,745	63,3035
	100	100,23	99,372	97,05	94,449	100	95,4494	97,182	100,55	64,5425
	100	102,45	98,299	96,452	99,684	100	104,297	92,827	95,2973	64,056
	100	100,24	100,97	101,47	106,05	100	100,542	98,68	100,155	62,1177
	100	101,84	103,05	101,62	86,577	100	103,408	102,51	102,215	60,8711
	100	98,924	102,28	100,88	81,781	100	101,087	90,819	102,077	85,2867
	100	100,39	103,23	94,395	83,83	100	90,834	87,476	93,5666	70,752
	100	100,39	98,428	99,153	89,792	100	97,7885	91,765	100,633	80,752
	100	101,46	104,99	99,659	97,637	100	99,4788	93,44	91,7647	60,0074
	100	100,41	100,78	98,235	95,442	100	98,3023	90,097	96,6121	83,2911
Mean	100	99,133	99,688	98,848	90,483	100	100,201	88,95	100,35	76,8206
SD	0	3,0811	3,6583	3,4465	7,1983	100	97,8667	94,161	97,9909	68,1871
SE	0	0,7473	0,8873	0,8359	1,7458	0	4,61441	4,375	3,84392	8,0958
%SE	0	0,7538	0,89	0,8456	1,9295	0	1,08763	1,0312	0,90602	1,9082
T.Test		0,2688	0,1042	0,1907	9E-06	0	1,11134	1,0951	0,9246	2,79848
							0,05806	2E-06	0,03337	6,2E-18
								**		***

48H

	100	111,71	84,792	84,938	52,535
	100	105,1	82,502	80,073	52,426
	100	104,04	68,773	81,196	51,119
	100	100,12	70,019	81,288	46,15
	100	103,65	73,01	80,488	52,603
	100	102,06	82,869	82,472	54,007
	100	93,218	92,104	93,316	66,519
	100	89,771	91,127	94,097	65,557
	100	95,256	91,301	94,4	65,807
	100	95,892	96,112	92,013	58,655
	100	95,218	93,354	97,309	59,239
	100	98,453	98,256	97,218	66,966
Mean	100	99,54	85,352	88,234	57,632
SD	0	6,103	10,169	7,0326	7,1678
SE	0	1,7618	2,9356	2,0301	2,0692
%SE	0	1,7699	3,4394	2,3009	3,5903
T-Test		0,7966	5E-05	8E-06	3E-13
			**	**	***
