# Supplementary material for: Genotoxicity and oxidative stress induction by polystyrene nanoparticles in the colorectal cancer cell line HCT116
Source: PLoS One. 2021 Jul 23;16(7):e0255120. doi: 10.1371/journal.pone.0255120 (PMC8301662; doi:10.1371/journal.pone.0255120)
Supplement: S2 File — ROS detection in HCT116 cells treated with Polystyrene Nanoparticles. (PDF) [file pone.0255120.s002.pdf]

ROS DATA ANALYSIS

These present data value deriving by technical triplicate of two biological sperimental duplicate.

T0

	Blank	Ctrl -	100	200	400	800	1200	Ctrl +
	12899	13303	13300	13220	13215	13245	13100	13502
	12883	12892	13426	12971	13080	13115	12914	13735
	12496	13190	13288	12734	13353	13019	13106	13279
	12698	13457	13578	12770	13133	13247	12873	13493
	12557	13311	13281	13244	13128	13463	13240	13405
	12763	12983	13425	12720	13397	12848	13381	13567
Mean	12716	13189	13383	12943	13218	13156	13102	13497
Dv St	165,886708	214,638922	116,5813021	241,43439	130,1102097	212,492274	192,19226	153,344601
Error St	67,7229651	87,6259728	47,59411728	98,565179	53,11727068	86,7496077	78,462164	62,60267122
T-Test vs ctrl			0,080791353	0,091527	0,787788531	0,79341048	0,4765079	0,017091572

T15 '

	Blank	Ctrl -	100	200	400	800	1200	Ctrl +
	12757	13154	13415	13171	13404	17787	12885	51685
	13288	12960	13038	12897	13864	18082	13228	47265
	12701	12742	13364	12941	13863	16991	12844	49601
	12653	13322	13550	13121	14105	18358	13205	33906
	12813	13472	13346	13300	14071	17567	13582	48280
	12718	13232	13449	13189	14357	16963	13242	49800
Mean	12822	13147	13360	13103	13944	17625	13164	46756
Dev St	234,725087	261,731924	173,7477098	154,81139	321,6146763	568,4972	270,85987	6470,844749
Error St	95,8261157	106,851611	70,9322055	63,201486	131,2986418	232,08801	110,57808	2641,711307
T-Test vs ctrl			0,127211477	0,7313543	0,000831288	7,7761E-09	0,9124805	1,69577E-07

T30'

	Blank	Ctrl -	100	200	400	800	1200	Ctrl +
	13023	12758	13137	13424	13179	18209	13089	84250
	13314	13120	13063	13349	13659	18672	13007	73011
	12701	13136	13418	12775	13972	17552	12884	76049
	12814	13000	13394	13092	14149	18826	13148	61929
	12659	13248	12949	13411	13869	18200	13503	74946
	12920	12751	13346	13380	14154	17667	13315	76185
Mean	12905	13002	13218	13239	13830	18188	13158	74395
Dev St	241,595875	207,33395	195,0932256	257,95096	369,178367	513,385106	221,9961	7220,540894
Error St	98,6311028	84,6437305	79,64647582	105,30804	150,7164372	209,588592	90,629527	2947,773476
T-Test vs ctrl			0,093184623	0,1108225	0,000733473	5,5909E-10	0,2383842	1,44958E-09

T45'

	Blank	Ctrl -	100	200	400	800	1200	Ctrl +
	13221	12858	13218	13147	13225	19059	13067	114519
	13472	12764	13163	13077	14163	19491	13014	97042
	12843	13384	13292	12765	14001	18037	13006	100098
	13042	12908	13103	12961	14141	19489	13314	94413
	13265	13209	13132	13096	13858	18526	13321	99737
	13193	13398	13300	12878	14384	18221	13136	98102
Mean	13173	13087	13201	12987	13962	18804	13143	100652
Dev St	212,898724	278,791977	82,67930011	146,57512	401,5051681	634,299745	142,94055	7097,852504
Error St	86,9155401	113,816348	33,75368293	59,839043	163,9137985	258,951787	58,355234	2897,686151
T-Test vs ctrl			0,357545802	0,4569583	0,001365389	1,9364E-09	0,6699036	3,71486E-11

T1h

	Blank	Ctrl -	100	200	400	800	1200	Ctrl +
	13557	13001	13254	13493	13640	19680	13132	140791
	13704	12955	13242	13102	14106	20379	13195	117909
	13092	13003	13414	13282	14450	18656	12884	122739
	13191	12965	13567	13324	14602	20271	13378	121569
	13407	13129	13285	13261	14417	19568	13734	121069
	13715	13020	13494	13306	14483	19030	13262	118147
Mean	13444	13012	13376	13295	14283	19597	13264	123704
Dev St	261,950886	62,3102453	136,3598181	125,4905	355,6357687	675,351661	283,01408	8589,068844
Error St	106,941001	25,4380511	55,66866264	51,231284	145,1876946	275,711161	115,54001	3506,472672
T-Test vs ctrl			0,000142293	0,0005881	6,07772E-06	3,9219E-10	0,0590165	2,39269E-11

T0

Blank	Ctrl -	100	200	400	800	1200	Ctrl +
	587	584	504	499	529	384	786
	176	710	255	364	399	198	1019
	474	572	18	637	303	390	563
	741	862	54	417	531	157	777
	595	565	528	412	747	524	689
	267	709	4	681	132	665	851
MEAN	473	667	227	502	440	386	781
Value %	1	1,40915493	0,4799296	1,059859155	0,929929577	0,816197183	1,64964789
SD	214,6389216	116,581302	241,43439	130,1102097	212,4922744	192,1922648	153,344601
SE	95,98944386	47,5941173	107,97274	58,18705469	86,74960775	85,95099379	62,6026712
T-Test vs ctrl		0,08079135	0,091527	0,787788531	0,793410476	0,476507887	0,01709157

T15 '

Blank	Ctrl -	100	200	400	800	1200	Ctrl +
	438	699	455	688	5071	169	38969
	244	322	181	1148	5366	406	34549
	26	648	225	1147	4275	128	36885
	606	834	405	1389	5642	489	21190
	756	630	584	1355	4851	866	35564
	410	733	367	1641	4247	420	37084
MEAN	413	627	370	1145	5041	413	33431
Value %	1	1,51596774	0,8939516	2,771129032	12,19596774	0,999327957	80,8824194
SD	258,4033024	173,74771	148,99631	321,6146763	568,4972002	265,2694577	6470,84475
SE	115,5614699	70,9322055	66,633175	131,2986418	232,0880101	132,6347288	2641,71131
T-Test vs ctrl		0,09923811	0,7263638	0,000684687	7,32503E-09	0,998570148	1,687E-07

T30'

Blank	Ctrl -	100	200	400	800	1200	Ctrl +
	42	421	708	463	5493	373	71534
	215	347	444	943	5956	291	60295
	231	702	59	1256	4836	168	63333
	284	678	376	1433	6110	432	49213
	532	233	695	1153	5484	787	62230
	35	630	475	1438	4951	410	63469
MEAN	223	502	459	1114	5472	410	61679
Value %	1	2,24869305	2,0588748	4,993278566	24,51829724	1,837814289	276,380881
SD	182,9944444	195,093226	238,77201	369,178367	513,3851056	208,1859323	7220,54089
SE	91,49722218	79,6464758	119,386	150,7164372	209,5885917	93,10357932	2947,77348
T-Test vs ctrl		0,02877087	0,0832429	0,000348662	4,25293E-10	0,129478714	1,4338E-09

T45'

Blank	Ctrl -	100	200	400	800	1200	Ctrl +
	142	502	431	509	6343	351	101803
	48	447	361	1447	6775	298	84326
	211	576		1285	5321	290	87382
	192	387	245	1425	6773	598	81697
	493	416	380	1142	5810	605	87021
	227	584	162	1668	5505	420	85386
MEAN	219	485	316	1246	6088	427	87936
Value %	1	2,21782178	1,4431074	5,693830922	27,81949733	1,951256664	401,839299
SD	149,0656455	82,6793001	109,66175	401,5051681	634,2997451	142,9405471	7097,8525
SE	74,53282275	33,7536829	49,042227	163,9137985	258,9517866	58,35523398	2897,68615
T-Test vs ctrl		0,00332111	0,2592963	0,000156303	8,20164E-10	0,033163803	3,6318E-11

T1h

Blank	Ctrl -	100	200	400	800	1200	Ctrl +
	285	538	777	924	6964	416	128075
	239	526	386	1390	7663	479	105193
	287	698	566	1734	5940	168	110023
	249	851	608	1886	7555	662	108853
	413	569	545	1701	6852	1018	108353
	304	778	590	1767	6314	546	105431
MEAN	296	636	579	1527	6995	548	112099
Value %	1	2,1487901	1,9538548	5,155880698	23,61778278	1,850872257	378,501069
SD	62,31024528	136,359818	125,4905	355,6357687	675,3516615	283,014075	8589,06884
SE	31,15512264	55,6686626	62,745252	145,1876946	275,7111613	126,5677421	4294,53442
T-Test vs ctrl		0,00014229	0,0005881	6,07772E-06	3,9219E-10	0,059016473	2,3927E-11

0'	Ctrl -	100 µg/ml	200 µg/ml	400 µg/m	800 µg/m	1200 µg/ı	Ctrl +
**MEAN**	473,2	667	227	502	440,1667	385,6	781
Value %	1	1,409552	0,4797126	1,061285	0,930192	0,814877	1,64792899
SD	239,9733	116,5813	240,67031	145,4603	212,4923	214,8681	171,421119
SE	107,3193	47,594117	107,63104	65,05183	86,74961	96,09194	69,9823787
SE%	0,226795	0,0713555	0,4741455	0,129585	0,197084	0,249201	0,08960612
T-Test vs ctrl		0,1126234	0,2217449	0,82305	0,813959	0,559977	0,04856202

15'	Ctrl -	100 µg/ml	200 µg/ml	400 µg/m	800 µg/m	1200 µg/ı	Ctrl +
**MEAN**	413	626,6	370	1145,4	5041	413	33431,4
Value %	1	1,5135266	0,8937198	2,766667	12,17633	0,997585	80,7521739
SD	288,8979	173,74771	166,57731	321,6147	568,4972	342,4159	6470,84475
SE	129,1991	70,932206	74,495637	131,2986	232,088	171,208	2641,71131
SE%	0,312831	0,1132017	0,2013396	0,114631	0,04604	0,414547	0,07901887
T-Test vs ctrl		0,1356463	0,7754828	0,001793	6,62E-08	0,996332	1,1018E-06

30'	Ctrl -	100 µg/ml	200 µg/ml	400 µg/m	800 µg/m	1200 µg/ı	Ctrl +
**MEAN**	223,25	501,83333	459,5	1114,333	5471,667	410,2	61679
Value %	1	2,2478537	2,0582307	4,991415	24,50915	1,837402	276,277716
SD	236,1544	195,09323	307,99621	369,1784	513,3851	232,7589	7220,54089
SE	118,0772	79,646476	153,99811	150,7164	209,5886	104,0929	2947,77348
SE%	0,528901	0,158711	0,3351428	0,135253	0,038304	0,253761	0,04779217
T-Test vs ctrl		0,0755258	0,2691434	0,002845	6,43E-08	0,272889	1,6932E-07

45'	Ctrl -	100 µg/ml	200 µg/ml	400 µg/m	800 µg/m	1200 µg/ı	Ctrl +
**MEAN**	218,75	485,33333	315,8	1246	6087,833	428,4	87935,8333
Value %	1	2,2186667	1,4436571	5,696	27,8301	1,9584	401,992381
SD	192,332	82,6793	109,66175	401,5052	634,2997	159,7664	7097,8525
SE	96,16598	33,753683	49,042227	163,9138	258,9518	65,22436	2897,68615
SE%	0,439616	0,0695474	0,1552952	0,131552	0,042536	0,152251	0,03295228
T-Test vs ctrl		0,0154419	0,3691754	0,001541	1,08E-07	0,116355	9,0325E-09

60'	Ctrl -	100 µg/ml	200 µg/ml	400 µg/m	800 µg/m	1200 µg/ı	Ctrl +
**MEAN**	296,5	636,4	579	1527	6994,8	548,6	112099,4
Value %	1	2,1463744	1,9527825	5,150084	23,59123	1,850253	378,075548
SD	80,13946	136,35982	161,70962	355,6358	675,3517	316,4171	8589,06884
SE	40,06973	55,668663	80,854808	145,1877	275,7112	141,506	4294,53442
SE%	0,135142	0,0874743	0,1396456	0,09508	0,039417	0,25794	0,03831006
T-Test vs ctrl		0,0014373	0,0203098	0,000125	6,03E-08	0,168732	6,4736E-09

DATI GRAFICO

	Ctrl -	100 µg/ml	200 µg/ml	400 µg/m	800 µg/m	1200 µg/ı	Ctrl +
0	1	1,409552	0,4797126	1,061285	0,930192	0,814877	1,64792899
15'	1	1,5135266	0,8937198	2,766667	12,17633	0,997585	80,7521739
30'	1	2,2478537	2,0582307	4,991415	24,50915	1,837402	276,277716

45'	1	2,2186667	1,4436571	5,696	27,8301	1,9584	401,992381
60'	1	2,1463744	1,9527825	5,150084	23,59123	1,850253	378,075548
SE%							
0	0,226795	0,0713555	0,5641258	0,129534	0,197084	0,249201	0,08974401
15'	0,312075	0,1132017	0,2013396	0,114631	0,04604	0,414547	0,07901887
30'	0,528901	0,158711	0,3351428	0,135253	0,038304	0,253761	0,04779217
45'	0,439616	0,0695474	0,1552952	0,131552	0,042536	0,152251	0,03295228
60'	0,135142	0,0874743	0,1396456	0,09508	0,039417	0,25794	0,03831006
