# Supplementary material for: Genotoxicity and oxidative stress induction by polystyrene nanoparticles in the colorectal cancer cell line HCT116
Source: PLoS One. 2021 Jul 23;16(7):e0255120. doi: 10.1371/journal.pone.0255120 (PMC8301662; doi:10.1371/journal.pone.0255120)

CBMN TEST DATA

FOR EACH CONDITION THREE SAMPLES HAVE BEEN MIXED

Glasses	Experim condit	Mononuclea	Binucleated	Multinucleated	Micronuclei	BUDs
SLIDE 1	Ctrl-	934	1000	11	3	13
SLIDE 2	Ctrl-	977	1000	13	3	13
SLIDE 3	Ctrl-	870	1000	12	3	12
SLIDE 1	800 µg/mL	766	1000	7	10	15
SLIDE 2	800 µg/mL	693	1000	5	9	16
SLIDE 3	800 µg/mL	713	1000	6	11	14
SLIDE 1	1200 µg/mL	742	1000	6	4	16
SLIDE 2	1200 µg/mL	700	1000	5	5	14
SLIDE 3	1200 µg/mL	789	1000	5	5	15
SLIDE 1	Ctrl+	786	1000	4	8	18
SLIDE 2	Ctrl+	838	1000	3	8	19
SLIDE 3	Ctrl+	882	1000	2	7	21

MONONUCLEA	Ctrl-	800 µg/mL	1200 µg/mL	Ctrl+
	934	766	742	786
	977	693	700	838
	870	713	789	882
MEAN	927	724	743,6667	835,3333333
SD	53,8423625	37,722672	44,5234	48,05552344
SE	31,08590249	21,779195	25,7056	27,7448694

BINUCLEATED	Ctrl-	800 µg/mL	1200 µg/mL	Ctrl+
	1000	1000	1000	1000
	1000	1000	1000	1000
	1000	1000	1000	1000
MEAN	1000	1000	1000	1000
SD	0	0	0	0
SE	0	0	0	0

ULTINUCLEATE	Ctrl-	800 µg/mL	1200 µg/mL	Ctrl+
	11	7	6	4
	13	5	5	3
	12	6	5	2
MEAN	12	6	5,333333	3
SD	1	1	0,57735	1
SE	0,577350269	0,5773503	0,333333	0,577350269

MICRONUCLEI	Ctrl-	800 µg/mL	1200 µg/mL	Ctrl+
	3	10	4	8
	3	9	5	8
	3	11	5	7
MEAN	3	10	4,666667	7,666666667
SD	0	1	0,57735	0,577350269
SE	0	0,5773503	0,333333	0,333333333

BUDs	Ctrl-	800 µg/mL	1200 µg/mL	Ctrl+
	13	15	16	18
	13	16	14	19
	12	14	15	21
MEAN	12,66666667	15	15	19,33333333
SD	0,577350269	1	1	1,527525232
SE	0,333333333	0,5773503	0,57735	0,881917104

CPBI (MONO+2*BI+3*MULTI)/TOTAL CELLS

Ctrl -	MONONUCLEAT	BINUCLEATE	MULTINUC	TOTAL CELLS	CBPI
	934	1000	11	1945	1,5254499
	977	1000	13	1990	1,5155779
	870	1000	12	1882	1,544102
MEAN				1939	1,5283766
SD					0,0144855
SE					0,0083632

800 µg/mL	MONONUCLEAT	BINUCLEATE	MULTINUC	TOTAL CELLS	CBPI
	766	1000	7	1773	1,571912
	693	1000	5	1698	1,5948174
	713	1000	6	1719	1,5887144
MEAN				1730	1,5851479
SD					0,0118619
SE					0,0068485

1200 µg/mL	MONONUCLEAT	BINUCLEATE	MULTINUC	TOTAL CELLS	CBPI
	742	1000	6	1748	1,5789474
	700	1000	5	1705	1,5923754
	789	1000	5	1794	1,5629877
MEAN				1749	1,5781035
SD					0,014712
SE					0,008494

Ctrl +	MONONUCLEAT	BINUCLEATE	MULTINUC	TOTAL CELLS	CBPI
	786	1000	4	1790	1,5631285
	838	1000	3	1841	1,5464422
	882	1000	2	1884	1,5329087
MEAN				1838,333333	1,5474931

SD	0,0151373
SE	0,0087395

GRAPH DATA

Micronuclei Frequency

		SE
Ctrl -	3	0
800 µg/mL	10	0,5773503
1200 µg/mL	4,666666667	0,3333333
Ctrl+	7,666666667	0,3333333

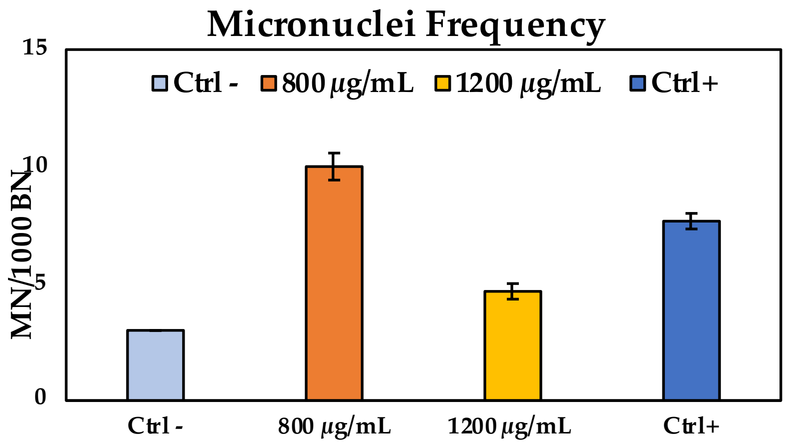

Nuclear BUDs Frequency

		SE
Ctrl -	12,66666667	0,3333333
800 µg/mL	15	0,5773503
1200 µg/mL	15	0,5773503
Ctrl+	19,33333333	0,8819171

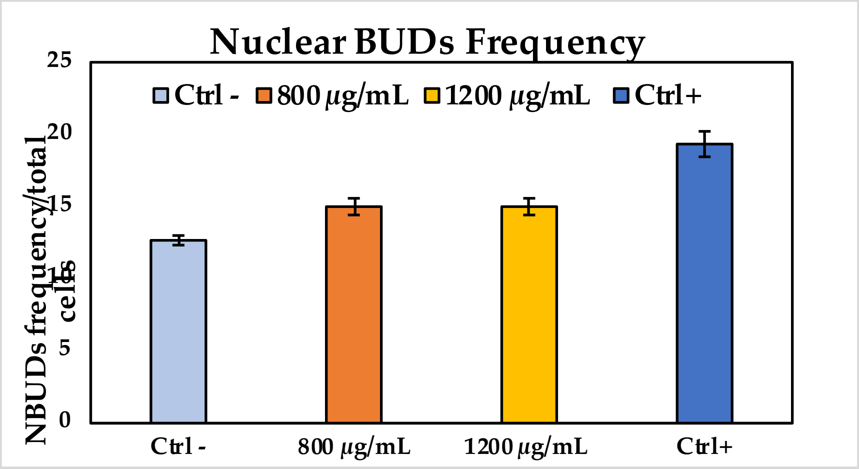

CPBI	
**Ctrl -**	1,544102
**800 µg/mL**	1,5851479
**1200 µg/mL**	1,5781035
**Ctrl+**	1,5474931
SE	0,0083632
SE	0,0068485
SE	0,008494
SE	0,0087395

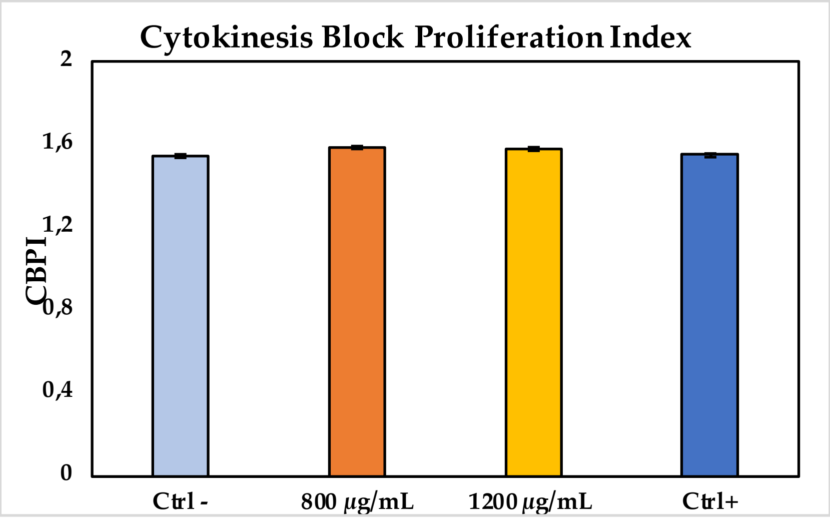

Supplement: S3 File — CBMN test in HCT116 cells treated with Polystyrene Nanoparticles. (PDF) [file pone.0255120.s003.pdf]
