# Supplementary material for: Genotoxicity and oxidative stress induction by polystyrene nanoparticles in the colorectal cancer cell line HCT116
Source: PLoS One. 2021 Jul 23;16(7):e0255120. doi: 10.1371/journal.pone.0255120 (PMC8301662; doi:10.1371/journal.pone.0255120)
Supplement: S4 File — Western Blot on the HCT116 cells treated with Polystyrene Nanoparticles. (PDF) [file pone.0255120.s004.pdf]

**1 Replicate**

| | area | pixel | | |
|---|---|---|---|---|
| **ACTIN** | area | pixel | | |
| 24H | | | | |
| Ctrl | 10580 | 30.805 | | |
| 800 ug/ml | 10580 | 39.618 | | |
| 1200ug/ml | 10580 | 41.615 | | |
| 48H | | | | |
| Ctrl | 9016 | 40.916 | | |
| 800 ug/ml | 10304 | 49.316 | | |
| 1200ug/ml | 10304 | 56.064 | | |
| | | | | |
| **CATALASE** | | | **Relative Intensity (arbitrary unit)** | |
| 24H | | | | |
| Ctrl | 6732 | 47.563 | 1,544003 | 1 |
| 800 ug/ml | 6732 | 33.490 | 0,845323 | 0,547488 |
| 1200ug/ml | 8250 | 42.550 | 1,022468 | 0,662219 |
| 48H | | | | |
| Ctrl | 6402 | 26.407 | 0,645395 | 1 |
| 800 ug/ml | 7722 | 25.419 | 0,515431 | 0,798628 |
| 1200ug/ml | 7722 | 22.920 | 0,408818 | 0,633439 |
| | | | | |
| **SOD1** | | | | |
| 24H | | | | |
| Ctrl | 13268 | 26.379 | 0,856322 | 1 |
| 800 ug/ml | 12198 | 28.165 | 0,710914 | 0,830195 |
| 1200ug/ml | 12198 | 26.615 | 0,639553 | 0,74686 |
| 48H | | | | |
| Ctrl | 12198 | 25.226 | 0,616531 | 1 |
| 800 ug/ml | 12198 | 24.004 | 0,486739 | 0,789479 |
| 1200ug/ml | 12198 | 24.880 | 0,443779 | 0,719799 |
| | | | | |
| **SOD2** | | | | |
| 24H | | | | |
| Ctrl | 9348 | 70.675 | 2,29427 | 1 |
| 800 ug/ml | 9348 | 61.684 | 1,556969 | 0,678634 |
| 1200ug/ml | 9348 | 60.680 | 1,458128 | 0,635552 |
| 48H | | | | |
| Ctrl | 9348 | 38.640 | 0,944374 | 1 |
| 800 ug/ml | 9348 | 59.339 | 1,20324 | 1,274114 |
| 1200ug/ml | 9348 | 63.758 | 1,137236 | 1,204222 |

## 2 REPLICATE

| ACTIN | area | pixel | | |
|---|---|---|---|---|
| 24H | | | | |
| Ctrl | 9202 | 108.739 | | |
| 800 ug/ml | 9202 | 116.977 | | |
| 1200ug/ml | 8272 | 122.324 | | |
| 48H | | | | |
| Ctrl | 9328 | 123.327 | | |
| 800 ug/ml | 9328 | 125.061 | | |
| 1200ug/ml | 9328 | 119.699 | | |
| | | | | |
| CATALASE | | | Relative Intensity | |
| 24H | | | | |
| Ctrl | 11881 | 12.133 | 0,111579 | 1 |
| 800 ug/ml | 10355 | 12.800 | 0,109423 | 0,980678 |
| 1200ug/ml | 10355 | 21.138 | 0,172803 | 1,548707 |
| 48H | | | | |
| Ctrl | 10355 | 17.131 | 0,138907 | 1 |
| 800 ug/ml | 10355 | 25.488 | 0,203805 | 1,4672 |
| 1200ug/ml | 10355 | 20.221 | 0,168932 | 1,216151 |
| | | | | |
| SOD1 | | | | |
| 24H | | | | |
| Ctrl | 11130 | 20.135 | 0,185168 | 1 |
| 800 ug/ml | 11130 | 22.386 | 0,191371 | 1,033498 |
| 1200ug/ml | 11130 | 15.480 | 0,126549 | 0,683428 |
| 48H | | | | |
| Ctrl | 11130 | 18.751 | 0,152043 | 1 |
| 800 ug/ml | 11130 | 14.599 | 0,116735 | 0,767777 |
| 1200ug/ml | 11130 | 15.216 | 0,127119 | 0,836072 |
| | | | | |
| SOD2 | | | | |
| 24H | | | | |
| Ctrl | 8118 | 37.080 | 0,341 | 1 |
| 800 ug/ml | 8118 | 28.594 | 0,244441 | 0,716836 |
| 1200ug/ml | 8118 | 25.534 | 0,208741 | 0,612143 |
| 48H | | | | |
| Ctrl | 8118 | 31.682 | 0,256894 | 1 |
| 800 ug/ml | 8118 | 23.535 | 0,188188 | 0,732551 |
| 1200ug/ml | 8118 | 34.973 | 0,292175 | 1,137334 |

**3 REPLICATE**

| | ACTIN | area | pixel |
|---|---|---|---|
| 24 H | Ctrl | 12.296 | 19.305 |
| | 800 ug/ml | 12.296 | 26.128 |
| | 1200ug/ml | 12.296 | 28.998 |
| 48H | Ctrl | 12.296 | 31.572 |
| | 800 ug/ml | 12.296 | 28.141 |
| | 1200ug/ml | 12.296 | 37.548 |

| | CATALASE | | | Relative Intensity | |
|---|---|---|---|---|---|
| 24 H | Ctrl | 12625 | 22.423 | 1,161513 | 1 |
| | 800 ug/ml | 12625 | 11.509 | 0,440485 | 0,379234 |
| | 1200ug/ml | 12625 | 11.406 | 0,393337 | 0,338642 |
| 48H | Ctrl | 12625 | 14.335 | 0,454042 | 1 |
| | 800 ug/ml | 12625 | 20.672 | 0,734587 | 1,617884 |
| | 1200ug/ml | 12625 | 32.916 | 0,876638 | 1,930744 |

| | SOD1 | | | | |
|---|---|---|---|---|---|
| 24 H | Ctrl | 12138 | 19.871 | 1,029319 | 1 |
| | 800 ug/ml | 12138 | 17.304 | 0,662278 | 0,643414 |
| | 1200ug/ml | 12138 | 25.207 | 0,869267 | 0,844507 |
| 48H | Ctrl | 12138 | 25.903 | 0,820442 | 1 |
| | 800 ug/ml | 12138 | 27.324 | 0,970968 | 1,183469 |
| | 1200ug/ml | 12138 | 44.134 | 1,175402 | 1,432645 |

| | SOD2 | | | | |
|---|---|---|---|---|---|
| 24 H | Ctrl | 10908 | 49.405 | 2,559182 | 1 |
| | 800 ug/ml | 10908 | 28.862 | 1,104639 | 0,431637 |
| | 1200ug/ml | 10908 | 36.210 | 1,248707 | 0,487932 |
| 48H | Ctrl | 10908 | 49.982 | 1,583112 | 1 |
| | 800 ug/ml | 10908 | 68.880 | 2,447674 | 1,546116 |
| | 1200ug/ml | 10908 | 58.544 | 1,559178 | 0,984882 |

| | GSPx | | | Actin | | |
|---|---|---|---|---|---|---|
| 24 H | Ctrl | 4896 | 27.080 | Ctrl | 6540 | 53.429 |
| | 800 ug/ml | 4896 | 18.829 | 800 ug/ml | 6540 | 47.987 |
| | 1200ug/ml | 4896 | 16.888 | 1200ug/ml | 6540 | 44.320 |
| 48H | Ctrl | 4896 | 26.481 | Ctrl | 6540 | 54.390 |
| | 800 ug/ml | 4896 | 29.290 | 800 ug/ml | 6540 | 55.641 |
| | 1200ug/ml | 4896 | 40.019 | 1200ug/ml | 6540 | 58.976 |

**Relative Intensity**

| | |
|---|---|
| 0,506841 | 1 |
| 0,392377 | 0,774162 |
| 0,381047 | 0,751808 |
| 0,486873 | 1 |
| 0,52641 | 1,081208 |
| 0,678564 | 1,39372 |

## 4 REPLICATE

| | ACTIN | area | pixel | | |
|---|---|---|---|---|---|
| 24H | Ctrl | 14637 | 15.102 | | |
| | 800 ug/ml | 14637 | 12.877 | | |
| | 1200ug/ml | 14637 | 10.889 | | |
| 48H | Ctrl | 14637 | 12.462 | | |
| | 800 ug/ml | 14637 | 6.604 | | |
| | 1200ug/ml | 14637 | 27.381 | | |
| | | | | Relative Intensity | |
| | CATALASE | | | | |
| 24H | Ctrl | 11400 | 20.971 | 1,388624 | 1 |
| | 800 ug/ml | 11400 | 15.751 | 1,223189 | 0,880864 |
| | 1200ug/ml | 11400 | 13.572 | 1,246395 | 0,897576 |
| 48H | Ctrl | 11400 | 17.142 | 1,375542 | 1 |
| | 800 ug/ml | 11400 | 10.366 | 1,569655 | 1,141118 |
| | 1200ug/ml | 11400 | 37.096 | 1,354808 | 0,984927 |
| | | | | | |
| | SOD1 | | | | |
| 24H | Ctrl | 20305 | 13.866 | 0,918157 | 1 |
| | 800 ug/ml | 18135 | 7.733 | 0,600528 | 0,654058 |
| | 1200ug/ml | 15965 | 6.997 | 0,642575 | 0,699854 |
| 48H | Ctrl | 15965 | 9.620 | 0,771947 | 1 |
| | 800 ug/ml | 15965 | 4.647 | 0,703664 | 0,911545 |
| | 1200ug/ml | 15965 | 11.500 | 0,419999 | 0,544078 |
| | | | | | |
| | SOD2 | | | | |
| 24H | Ctrl | 13440 | 30.196 | 1,99947 | 1 |
| | 800 ug/ml | 11526 | 24.562 | 1,907432 | 0,953969 |
| | 1200ug/ml | 9690 | 23.278 | 2,137754 | 1,06916 |
| 48H | Ctrl | 11526 | 26.960 | 2,163377 | 1 |
| | 800 ug/ml | 10098 | 21.338 | 3,231072 | 1,493532 |
| | 1200ug/ml | 11639 | 29.838 | 1,089734 | 0,503719 |

| | GSPx | area | pixel | Actin | area | pixel | Relative Intensity | |
|---|---|---|---|---|---|---|---|---|
| 24H | Ctrl | 8400 | 65.414 | Ctrl | 7020 | 98700 | 0,662756 | 1 |
| | 800 ug/ml | 8400 | 60.227 | 800 ug/ml | 7020 | 93670 | 0,64297 | 0,970146 |
| | 1200ug/ml | 3456 | 61.666 | 1200ug/ml | 7020 | 98760 | 0,624403 | 0,971122 |
| 48H | Ctrl | 8400 | 74.630 | Ctrl | 7020 | 122.228 | 0,61058 | 1 |
| | 800 ug/ml | 8400 | 94.658 | 800 ug/ml | 7020 | 101.672 | 0,931013 | 1,524801 |
| | 1200ug/ml | 8400 | 107.264 | 1200ug/ml | 7020 | 100.615 | 1,066084 | 1,746017 |

## MEDIATED VALUES

| | | | | | | | Values Mediated | SD | SE |
|---|---|---|---|---|---|---|---|---|---|
| | Catalase | | | | | | | | |
| | Ctrl | 1 | 1 | 1 | | Ctrl | 1 | 0 | 0 |
| 24H | 800 ug/ml | 0,547488 | 0,98067844 | 0,8808638 | | 800 | 0,803010066 | 0,2268466 | 0,13097 |
| | 1200ug/ml | 0,762219 | 1,34870734 | 0,8975759 | | 1200 | 1,002834078 | 0,3070857 | 0,177296 |
| 48H | Ctrl | 1 | 1 | 1 | 1 | Ctrl | 1 | 0 | 0 |
| | 800 ug/ml | 0,7986283 | 1,46719998 | 1,4111758 | 1,6179 | 800 | 1,32372202 | 0,3708866 | 0,2141315 |
| | 1200ug/ml | 0,6334388 | 1,21615116 | 0,984927 | 1,9307 | 1200 | 1,191315168 | 0,2934173 | 0,1694046 |
| | | | | | | | | | |
| | SOD1 | | | | | | | | |
| 24H | Ctrl | 1 | 1 | 1 | 1 | Ctrl | 1 | 0 | 0 |
| | 800 ug/ml | 0,830195 | 1,03349819 | 0,6540585 | 0,6434 | 800 | 0,790291378 | 0,1898819 | 0,1096283 |
| 48H | 1200ug/ml | 0,7468604 | 0,68342834 | 0,6998535 | 0,8445 | 1200 | 0,743662296 | 0,0329218 | 0,0190074 |
| | Ctrl | 1 | 1 | 1 | 1 | Ctrl | 1 | 0 | 0 |
| | 800 ug/ml | 0,7894789 | 0,76777673 | 0,9115454 | 1,1835 | 800 | 0,913067435 | 0,0775033 | 0,0447466 |
| | 1200ug/ml | 0,7197987 | 0,83607206 | 0,5440781 | 1,4326 | 1200 | 0,883148396 | 0,1470021 | 0,0848717 |
| | | | | | | | | | |
| | SOD2 | | | | | | | | |
| 24H | Ctrl | 1 | 1 | 1 | | Ctrl | 1 | 0 | 0 |
| | 800 ug/ml | 0,6786336 | 0,71683639 | 0,9539686 | | 800 | 0,783146206 | 0,1491646 | 0,0861202 |
| 48H | 1200ug/ml | 0,635552 | 0,61214286 | 1,06916 | | 1200 | 0,772284954 | 0,2573676 | 0,1485913 |
| | Ctrl | 1 | 1 | 1 | 1 | Ctrl | 1 | 0 | 0 |
| | 800 ug/ml | 1,2741144 | 0,73255103 | 1,4935319 | 1,5461 | 800 | 1,261578341 | 0,3916901 | 0,2261424 |
| | 1200ug/ml | 1,2042223 | 1,13733379 | 0,5037189 | 0,9849 | 1200 | 0,957539161 | 0,3865762 | 0,2231899 |
| | | | | | | | | | |
| | GSPx | | | | | | | | |
| 24H | Ctrl | 1 | 1 | | | Ctrl | 1 | 0 | 0 |
| | 800 ug/ml | 0,9701461 | 0,77416234 | | | 800 | 0,872154237 | 0,1385815 | 0,08001 |
| | 1200ug/ml | 0,9711224 | 0,75180785 | | | 1200 | 0,86146514 | 0,1550788 | 0,0895348 |
| 48H | Ctrl | 1 | 1 | | | Ctrl | 1 | 0 | 0 |
| | 800 ug/ml | 1,5248012 | 1,08120768 | | | 800 | 1,303004434 | 0,313668 | 0,1810963 |
| | 1200ug/ml | 1,7460172 | 1,39372021 | | | 1200 | 1,569868709 | 0,2491116 | 0,1438247 |
