# Supplementary figures and images for: Genotoxicity and oxidative stress induction by polystyrene nanoparticles in the colorectal cancer cell line HCT116
Source: PLoS One. 2021 Jul 23;16(7):e0255120. doi: 10.1371/journal.pone.0255120 (PMC8301662; doi:10.1371/journal.pone.0255120)

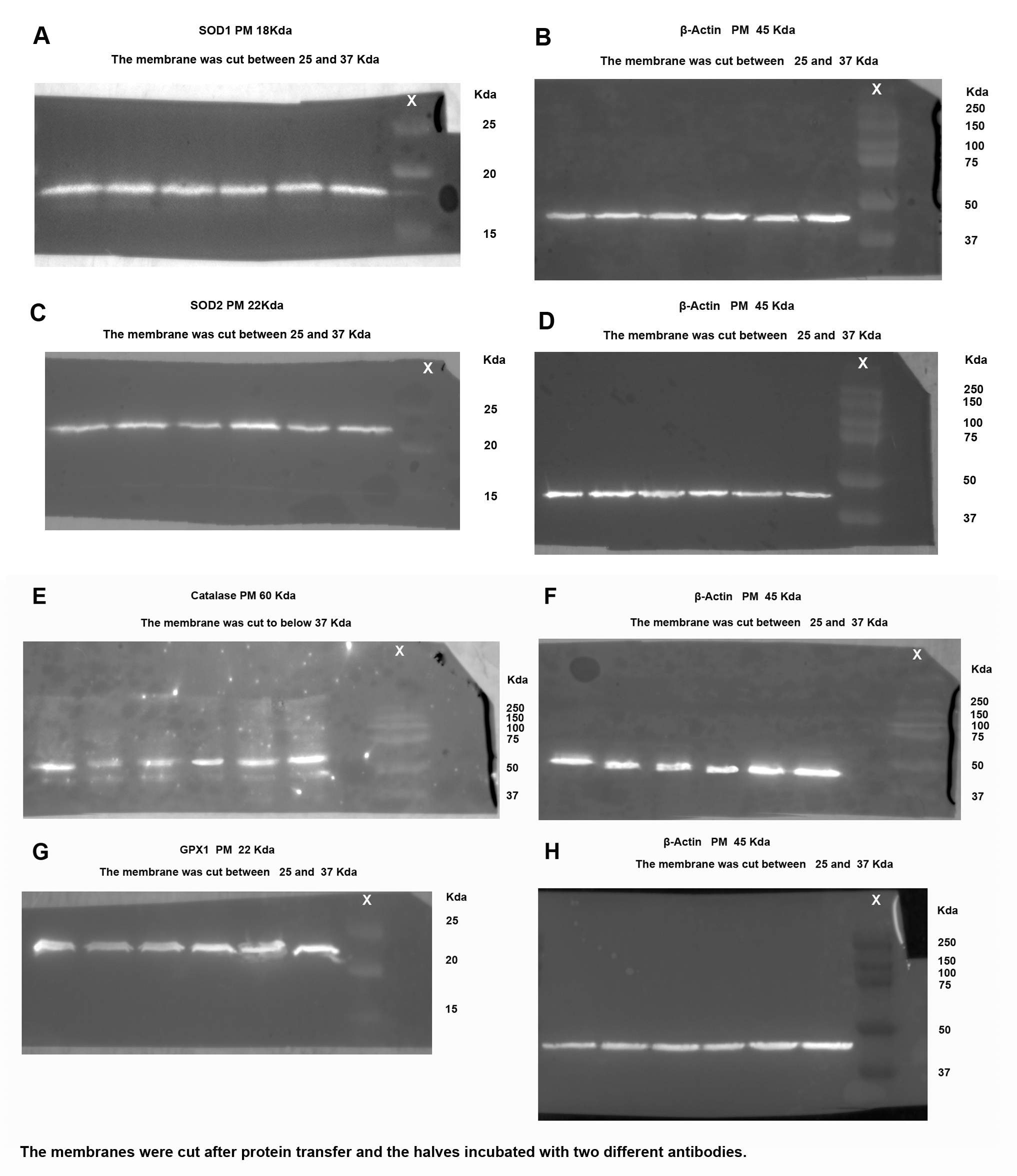

Supplement: S1 Raw images — Western Blot, SOD1 and 2 analysis. Western Blot Catalase and GPx1 analysis. (TIF) [file pone.0255120.s005.tif]
